# A new stem saurian reptile from the late Permian of South Africa and insights into saurian evolution

**DOI:** 10.1186/s13358-025-00351-y

**Published:** 2025-02-26

**Authors:** Ethan Dean Mooney, Diane Scott, Robert Raphael Reisz

**Affiliations:** 1 Department of Biology, University of Toronto Mississauga, 3359 Mississauga Rd., Mississauga, ON L5L1C6 Canada; 2 Dinosaur Evolution Research Center, International Center of Future Science, Jilin University, 2699 Qianjin Str., Changchun, 130012 Jilin China

**Keywords:** Diapsid, Neodiapsid, Younginiform, Paleozoic

## Abstract

The evolutionary radiation of diapsid reptiles that includes all extant and most extinct reptiles is well-represented in the Mesozoic and Cenozoic fossil records, however, the earliest stages recorded in the Paleozoic Era are limited to comparatively few taxa. Consequently, the origins of Sauria, the crown-group of Diapsida, remains poorly understood and the phylogenetic positions of the few known taxa along the saurian stem are controversial. Here, we describe *Akkedops bremneri* sp. et gen. nov., a new early late Permian stem saurian from the Karoo of South Africa based on two skulls and show that the famous aggregation of "juvenile *Youngina*” SAM-PK-K7710 is also referrable to it, thereby making this one of the best-known stem saurians. The skull has a short rostrum, open lower temporal bar, large contribution of the postfrontal to the upper temporal fenestra, slender stapes, sliver-like supratemporal with a distinct lateral flange suturing to the postorbital, and lacks both postparietal and tabular bones. The saddle-shaped quadrate is rather saurian-like in being posteriorly emarginated with a tympanic crest and unique medial flange. The post cranial skeleton of *Akkedops bremneri *is lizard-like and notably shows a hook-shaped fifth metatarsal and thyroid fenestra. Phylogenetic analysis recovers *Akkedops* *bremneri *as sister to Sauria, which is especially surprising considering its rather small size and slender, lizard-like morphology prior to the split between the apparently similar lepidosauromorphs and many of the comparatively robust archosauromorph saurians. Our analysis also indicates that *Youngina capensis* falls outside the clade of *Akkedops bremenri* + Sauria and does not appear to form a clade with other “younginiform” reptiles. The available evidence indicates a surprising level of complexity related to the evolution of stem saurians and the origin of Sauria that occurred in the shadow of other Paleozoic amniotes.

**Supplementary Information:**

The online version contains supplementary material available at 10.1186/s13358-025-00351-y.

## Introduction

The evolutionary history of Paleozoic diapsid reptiles is poorly represented in the fossil record, in strong contrast to the wonderfully diverse Mesozoic assemblages, which include those that form crown Sauria. Sauria includes a multitude of extinct taxa as well as extant crocodilians, birds, testudines, and lepidosaurs (snakes, lizards, and rhynchocephalians), making it the most diverse and speciose clade of amniotes (Ezcurra et al., 2014; Pincheira-Donoso et al., 2013; Roll et al., 2017; Simões et al., 2022). The origins of this prolific clade and that of its two dichotomous lizard and crocodilian lineages are poorly understood and the subject of debate. The earliest recognized non-archosaurian archosauromorphs and non-lepidosaurian lepidosaurs are limited to the late Permian and the earliest Triassic respectively (Bernardi et al., 2015; Carroll, 1975, 1977; Ezcurra et al., 2014; Ford et al., 2021; Jones et al., 2013; Simões et al., 2022). This limited record has been attributed to historic collection biases but also their relatively small size, rarity, and the poor preservational conditions of their apparently preferred upland environments in the early and middle Permian (Mooney et al., 2022; Reisz et al., 2011). As such, the phylogenetic relationships of many stem saurians remain without consensus (e.g., Brocklehurst et al., 2022; Buffa et al., 2024; Simões et al., 2022).

Only in the late Permian does the Paleozoic diapsid fossil record improve slightly, although it still demonstrates that these typically small, gracile bodied predators were the rarest members of their respective communities. The majority of these taxa are from the late Permian of Gondwanan and renewed interest in this pivotal episode of reptile evolution has prompted further study and reexamination of these peculiar reptiles (e.g., Buffa et al., 2021, 2022; Gardner et al., 2010; Hunt et al., 2023). Here, we reevaluate the anatomy of *Youngina capensis* and describe a new stem saurian based on several specimens from the same late Permian locality in South Africa, including an aggregation of individuals mistakenly identified as *Youngina capensis* (Smith & Evans, 1996).

## Methods

### Phylogenetic inference

Using the taxon and character dataset from Buffa et al. (2024), we reassess the phylogenetic relationships of late Permian neodiapsids by adding a new taxon (*Akkedops bremneri*) and by modifying several scorings of *Youngina* based on personal observations of the senior author. Our analysis consisted of 78 taxa with *Seymouria spp.* and *Limnoscelis paludis* as the outgroups and a total of 403 characters. This phylogenetic analysis was conducted under maximum parsimony in PAUP* v.4.0a.169 (Swofford, 1998) using a heuristic stepwise analysis with 1000 random additional sequence replicates. Branches lacking transformations under all optimizations (branch length = 0) were collapsed and branch support was gauged using Bremer support values and Bootstrap resampling analysis with 1000 pseudoreplications.

### Computed tomography and segmentation

Both SAM-PK-K7710 and the holotype SAM-PK-K6205 were scanned together for a duration of four hours using the μCT instrument at the Stellenbosch University Central Analytical Facility in South Africa (Plessis et al., 2016). All segmentation for production of 3D renderings was performed in the segmentation software Dragonfly v. 2021 using a non-commercial license and all rearticulation of the resulting 3D renderings was done in the freely available 3D imaging software Blender v.4.1.

### Institutional abbreviations

BP: Evolutionary Studies Institute (ESI: formerly Bernard Price Institute), University of the.

Witwatersrand, Johannesburg, South Africa.

SAM-PK: South African Museum, Cape Town, South Africa.

### Nomenclatural acts

The electronic edition of this article conforms to the requirements of the amended International Code of Zoological Nomenclature, and hence the new names contained herein are available under that Code from the electronic edition of this article. This published work and the nomenclatural acts it contains have been registered in ZooBank, the online registration system for the ICZN. The ZooBank LSIDs (Life Science Identifiers) can be resolved and the associated information viewed through any standard web browser by appending the LSID to the prefix “https://can01.safelinks.protection.outlook.com/?url=http%3A%2F%2Fzoobank.org% 2F&data = 05%7C01%7Crobert.reisz%40utoronto.ca%7Cd97a3dc3a47f44f90c9f08dabbf96cda %7C78aac2262f034b4d9037b46d56c55210%7C0%7C0%7C638028977364866541% 7CUnknown%7CTWFpbGZsb3d8eyJWIjoiMC4wLjAwMDAiLCJQIjoiV2luMzIiLCJBTiI6Ik 1haWwiLCJXVCI6Mn0%3D%7C3000%7C%7C%7C&sdata = rsST4QLtDFacXwh0TWmaI8 c92lduy%2BRXe8D8oVtSIr4%3D&reserved = 0”. The LSID for this publication is: urn:lsid:zoobank.org:pub:C50F5EFB-D181-4A77-9ACD-7FECAC948239. The electronic edition of this work was published in a journal with an ISSN, and has been archived and is available from the following digital repositories: PubMed Central, LOCKSS.

### Systematic paleontology

Reptilia Laurenti, 1768

Diapsida Osborn, 1903.

Neodiapsida Benton, 1985

*Akkedops* gen. nov.

urn:lsid:zoobank.org:act:D5DDDCC7-0E00-4038–8307-810E4B9278BE.

*Akkedops bremneri* sp. nov.

urn:lsid:zoobank.org:act:59219159-53B0-429D-A172-5B9C2B59CA68.

### Etymology

*Akkedops* = *Akkedis* (lizard) + ópsis, “appearance” *bremneri.*

The generic name from Afrikaans *akkedis* meaning “lizard”, for its lizard-like appearance, and the Greek *ópsis* for “appearance”.

The species name *bremneri*, honors the late D.T. Bremner, the amateur collector who found and donated BP/1/2614 and the holotype skull SAM-PK-K6205.

### Holotype

The holotype (SAM-PK-K6205) is an isolated and nearly complete skull with the posterior portion slightly compressed vertically and portions of the shoulder girdle positioned alongside the rostrum. The anterior most portion of the rostrum is slightly crushed, the premaxilla is incomplete, and the left lower orbital margin including the left jugal is fragmentary.

### Referred specimens

BP/1/2614 is an isolated and nearly complete crushed skull lacking the anterior most portion of the rostrum and mandible.

The famous aggregation of "juvenile *Youngina*” (SAM-PK-K7710) was originally described by Smith and Evans (1996). Reexamination of this specimen reveals five largely articulated individuals and likely the partial hindlimbs of two additional individuals. SAM-PK-K7710a, denoted in yellow, is a nearly complete individual although it lacks most of the tail and the skull appears to be mediolaterally compressed. It also exhibits the highest degree of lithostatic distortion of any skull in this aggregation of skeletons. SAM-PK-K7710b, denoted in blue, is another nearly complete individual although it too lacks much of the tail and the rostrum appears somewhat crushed. SAM-PK-K7710c, denoted in red, has a very well-represented skull and shoulder girdle, but it completely lacks the right forelimb and postcranium just posterior to the shoulder girdle. SAM-PK-K7710d, denoted in green, is articulated in an un-natural position where the body is bent nearly 90° at the pelvis, while the shoulder girdle and skull are largely disarticulated, which likely reflects some postmortem distortion and disarticulation. SAM-PK-K7710e, denoted in purple, lacks the skull and much of the shoulder girdle but is otherwise almost completely represented and it is articulated in a gently curled position beneath SAM-PK-K7710a-d. SAM-PK-K7710f, denoted in pink, represents two sets of articulated phalanges complete with unguals and a series of phalanges, all of which are spatially distinct from all other individuals of this aggregation and likely indicate a sixth individual. SAM-PK-K7710g, denoted in orange, is likely a partially articulated hindlimb consisting of what appears to be the fibula, disarticulated phalanges, distal section of the femur, and several caudal vertebrae.

### Locality and horizon

SAM-PK-K7710 was recovered by R.M.H. Smith from the *Endothiodon* Assemblage Zone (Day & Smith, 2020) (formerly the *Tropidostoma* Assemblage Zone [Smith & Keyser, 1995] as well as the lower *Cistecephalus* Zone [Kitching, 1977]) between the Beaufort West and Lozton districts of the Cape Province of South Africa. It was about 700 m lower in the Beaufort succession than strata typically containing *Youngina capensis* and has an estimated age of roughly 260 Ma (Day et al., 2015; Smith & Evans, 1996). The skulls SAM-PK-K6205 and BP/1/2614 were collected by D.T. Bremner in the 1980s from same site. The sedimentary environment has been interpreted as a muddy floodplain that experienced periodic flooding from a large river as part of a larger foreland basin environment (refer to Smith & Evans [1996] for further discussion on the geology of this locality).

### Diagnosis

A small lizard-like diapsid reptile distinguishable from other Permian diapsids by the following features: a saddle-shaped and posteriorly emarginated quadrate with a distinct medial process and tympanic crest, a sliver-like supratemporal with a distinct lateral flange that covers part of the head of the squamosal and sutures to the postorbital to make a small contribution to the upper temporal fenestra. It is also easily differentiable from *Youngina capensis* by the following features: homodont dentition of the maxilla consisting of at least twenty-six peg-like, non-serrated teeth with only very slight recurvature at the crown apex, a more dentulous palate, short broad nasal half the anteroposterior length of the frontal, larger contribution of the postfrontal to the upper temporal fenestra, short free-ending posterior process of the jugal, highly reduced quadratojugal, lack of a lower temporal bar, sloping occipital shelf of the parietal indicating lack of postparietal and tabular, presacral vertebrae with neural arch body transversely broader than respective centrum, presacral vertebrae with pedicel dorsoventrally taller than that of the respective centrum, posterior presacral vertebrae with double costal facets, and short forelimb with ulna and radius 70% the length of the humerus.

### Remarks

**Fig. 1:**
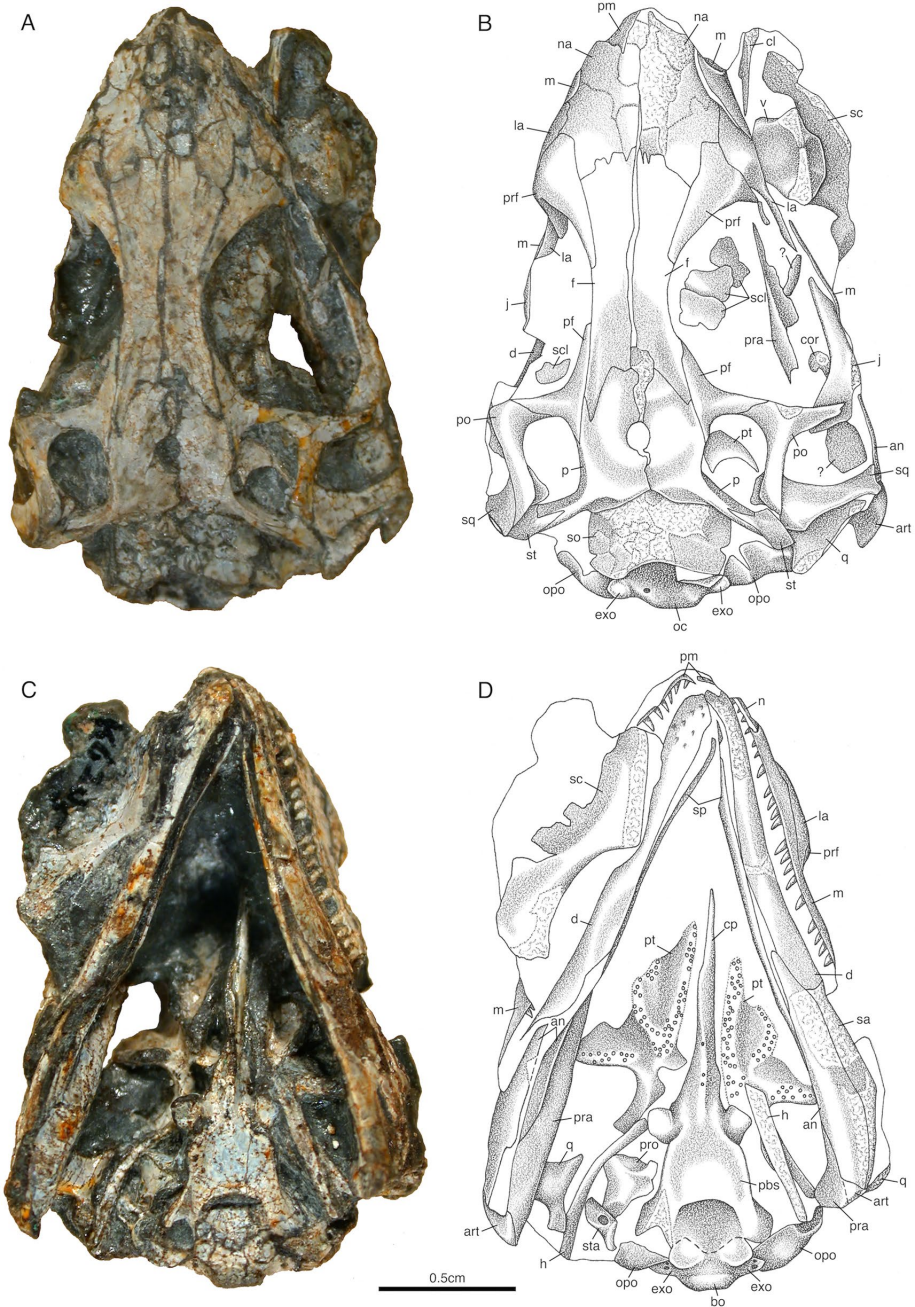
*Akkedops bremneri* photographs and illustrations of holotype skull SAM-PK-K6205. **A, B** dorsal view. **C, D** ventral view. *an* angular, *art* articular, *bo* basioccipital, *cl* clavicle, *cor* coronoid, *cp* cultriform process, *d* dentary, *exo* exoccipital, *f* frontal, *h* hyoid, *j* jugal, *la* lacrimal, *m* maxilla, *n* nasal, *oc* occipital, *opo* opisthotic, *p* parietal, *pbs* parabasisphenoid, *pf* postfrontal, *po* postorbital, *prf* prefrontal, *pro* prootic, *pt* pterygoid, *q* quadrate, *sa* surangular, *sc* scapula, *scl* scleral plate, *so* supraoccipital, *sp* splenial, *st* supratemporal, *sta* stapes, *sq* squamosal, *v* vertebra, *?* unknown

**Fig. 2:**
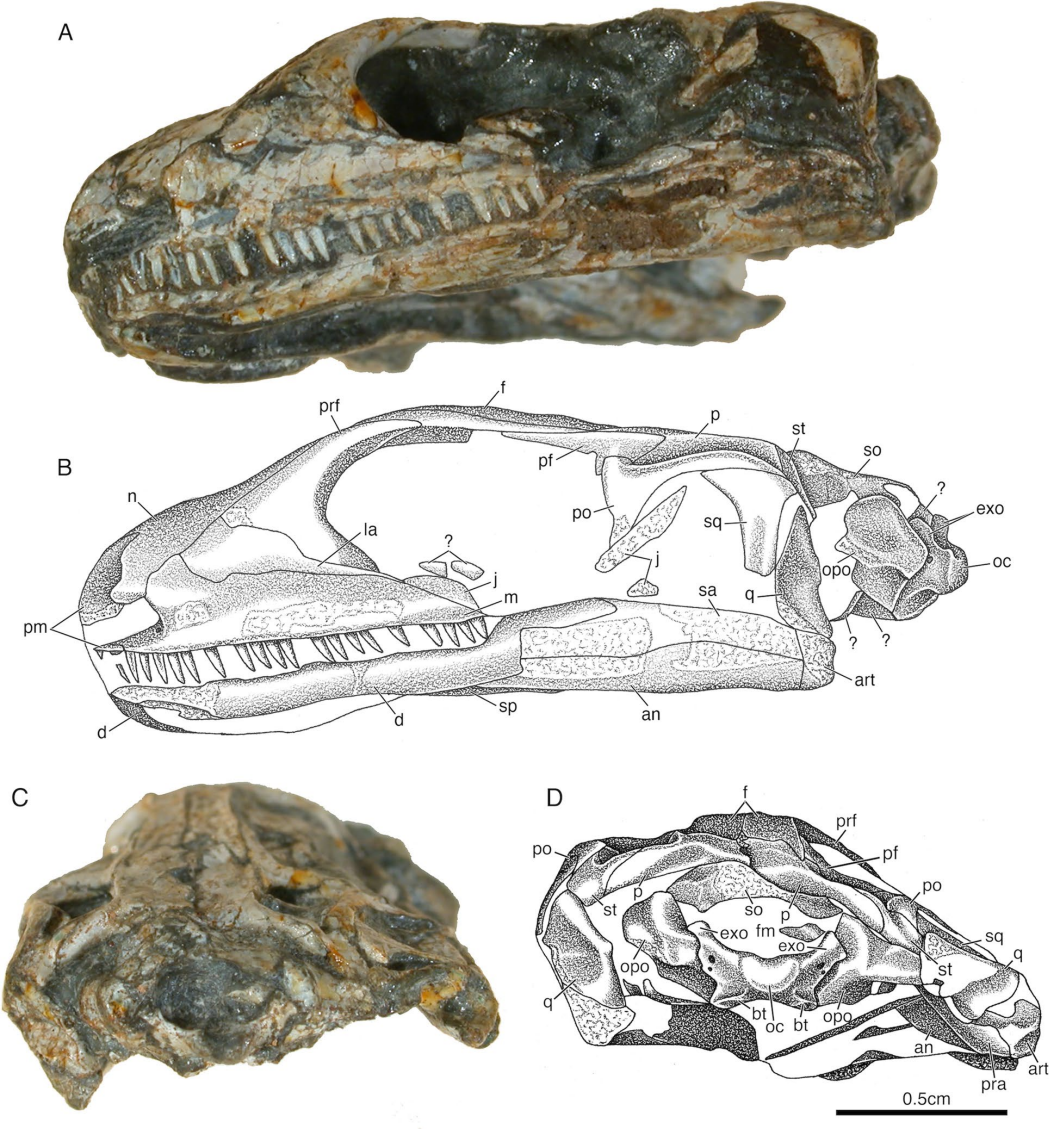
*Akkedops bremneri* photographs and illustrations of holotype skull SAM-PK-K6205 in left lateral and occipital views. **A** lateral view photograph is at a slightly different angle from **B,** the illustration. **C** occipital view photograph is similarly at a slightly different angle from **D**, the illustration. *an* angular, *art* articular, *bo* basioccipital, *bpp* basipterygoid process, *bt* basal tubera of basioccipital, *d* dentary, *exo* exoccipital, *f* frontal, *fm* foramen magnum, *j* jugal, *la* lacrimal, *m* maxilla, *n* nasal, *oc* occipital, *opo* opisthotic, *p* parietal, *pf* postfrontal, *po* postorbital, *prf* prefrontal, *q* quadrate, *so* supraoccipital, *sp* splenial, *st* supratemporal, *?* unknown

**Fig. 3:**
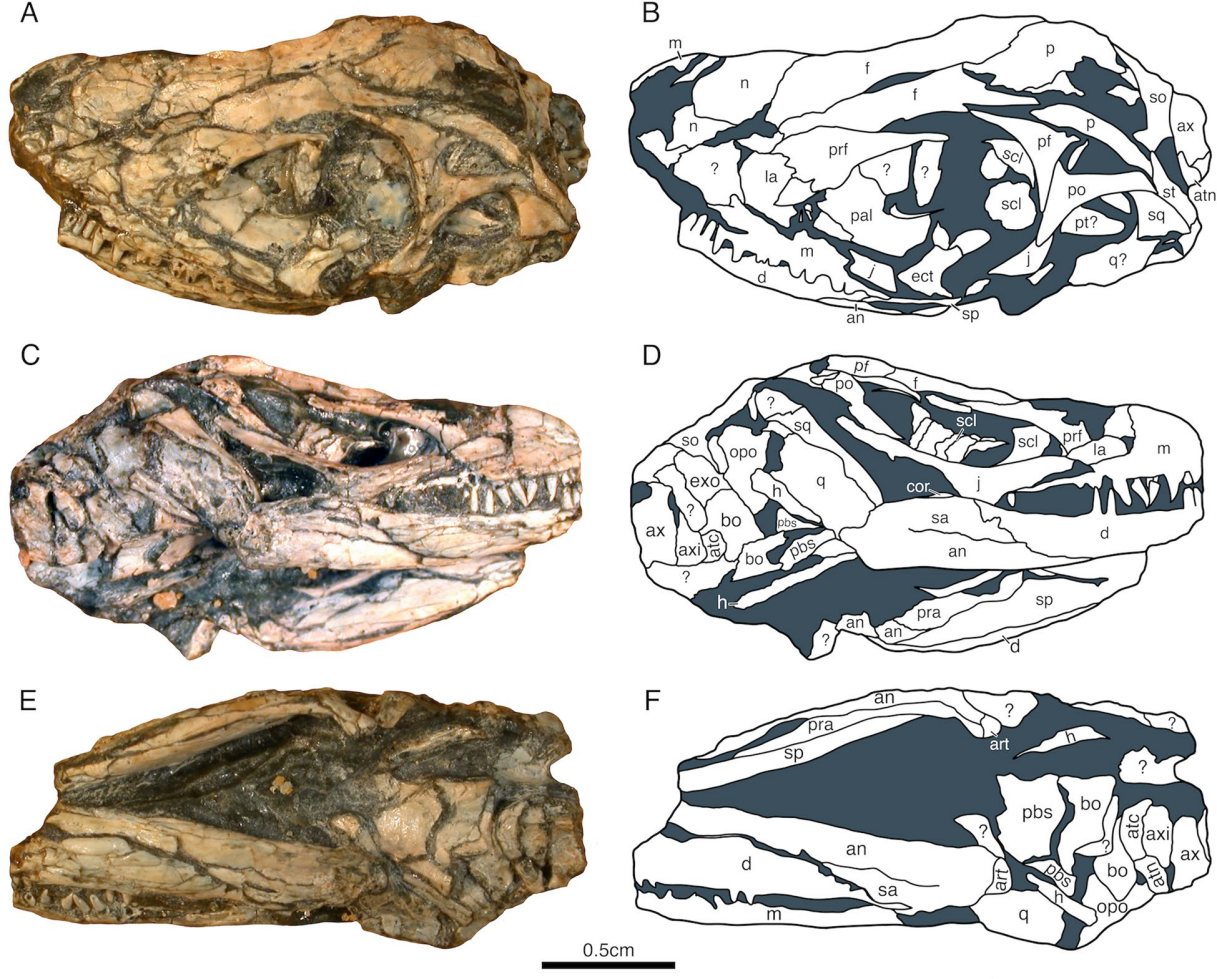
*Akkedops bremneri* skull BP/1/2614 photographs and outline illustrations. **A, B** left lateral view. **C, D** right lateral view. **E, F** ventral view. *an* angular, *art* articular, *atc* atlantal centrum, *atn* atlas neural arch, *axi* axis intercentrum, *bo* basioccipital, *cor* coronoid, *d* dentary, *exo* exoccipital, *f* frontal, *h* hyoid, *j* jugal, *la* lacrimal, *m* maxilla, *n* nasal, *oc* occipital, *opo* opisthotic, *p* parietal, *pbs* parabasisphenoid, *pf* postfrontal, *po* postorbital, *prf* prefrontal, *q* quadrate, *sa* surangular, *scl* scleral plate, *so* supraoccipital, *sp* splenial, *st* supratemporal, *sq* squamosal,* ?* unknown

**Fig. 4:**
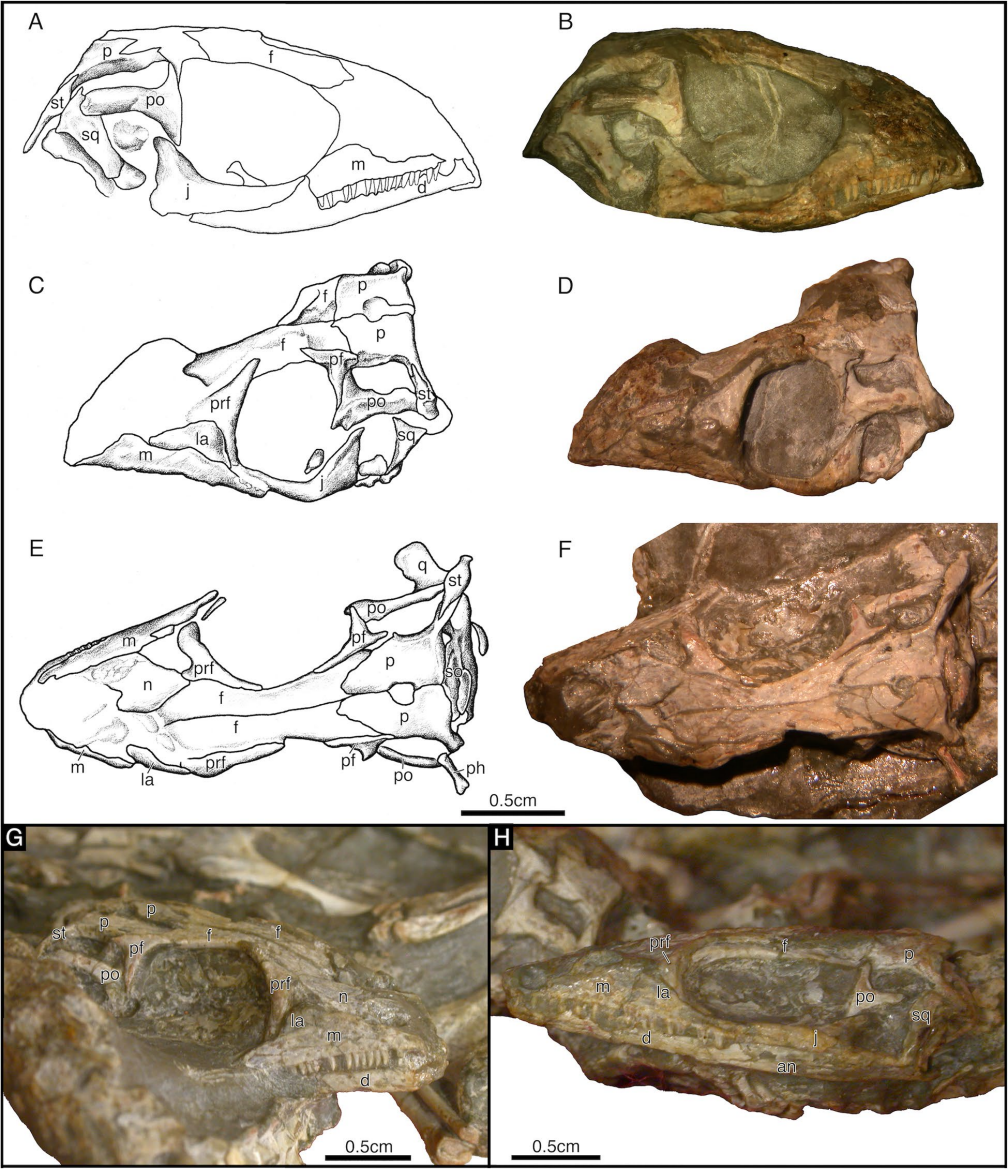
*Akkedops bremneri* skulls from SAM-PK-K7710 as photographs and illustrations. **A, B** right lateral view and **C**, **D** left lateral view of SAM-PK-K7710b skull. **E**, **F** dorsal view and **G** right lateral view of SAM-PK-K7710c. **H** left lateral view of SAM-PK-K7710a. *an* angular, *d* dentary, *f* frontal, *j* jugal, *la* lacrimal, *m* maxilla, *n* nasal, *p* parietal, *pf* postfrontal, *ph* phalange, *po* postorbital, *prf* prefrontal, *q* quadrate, *sa* surangular, *so* supraoccipital, *sp* splenial, *st* supratemporal, *sq* squamosal

The skulls SAM-PK-K6205, BP/1/2614, and those from the nearly complete skeletons SAM-PK-K7710a-d (Figs. 1, 2, 3 and 4) were all collected within close proximity to one another at the same locality and are anatomically indistinguishable from each other. *Akkedops bremneri* bears similarity to other late Permian terrestrial Gondwanan neodiapsids, mainly *Youngina capensis* (Gow, 1975) but also *Acerosodontosaurus piveteaui* (Bickelmann et al., 2009) and *Saurosternon bainii* (Carroll, 1975). However, most interestingly, it exhibits several anatomies suggestive of saurian affinities (see Discussion below).

SAM-PK-K7710 was originally interpreted by Smith and Evans (1996) as an aggregation of juvenile *Youngina capensis* largely because these individuals measure roughly half the size and have several skeletal similarities. The mediolaterally compressed skull of SAM-PK-K7710a superficially resembles the narrow rostrum of *Youngina capensis*. However, closer inspection of all individuals from this aggregation indicates they are all skeletally mature individuals referrable to the same taxon as the holotype of *Akkedops bremneri*.

The famous aggregation of "juvenile *Youngina*” SAM-PK-K7710 is reassigned to *Akkedops bremneri* and is distinguishable from *Youngina capensis* by the following currently observable features shared with the holotype SAM-PK-K6205: At least twenty-six proportionately smaller, nearly peg-like, non-serrated teeth of the maxilla with only modest distal recurvature of the crown (Fig. 4G); minor contribution of the lacrimal to the orbital margin (Fig. 4C, D, G, H; a lateral expansion of the supratemporal to contact the postorbital and contributes to the upper temporal fenestra (Fig. 4C, D, E, F; greater contribution of the postfrontal to the upper temporal fenestra (Fig. 4E, F, G; lack of a lower temporal bar (Fig. 4H); a more slender squamosal (Fig. 4C, D; sloping occipital shelf of the parietal lacking a tabular and postparietal (Fig. 4E, F); a smaller pineal foramen, roughly ¼ of the interparietal length (Fig. 4C, D, E, F. There are also several anatomies of the postcranial skeleton that differentiate SAM-PK-K7710 from *Youngina capensis* (see Discussion below).

No skeletal indicators suggest individuals of SAM-PK-K7710 are juvenile. On the contrary, the overall well-ossified nature of these skeletons, including the fusion of the neurocentral suture and well-developed distal articulating surfaces of the limb bones (e.g., the well-ossified tuberosity of the proximal articulating surface of the femur), suggests that this is an aggregation of subadult or adult individuals—perhaps representing an estivating behavior. Such well-defined articulating surfaces are also in strong contrast to the poorly ossified articulating surfaces of juvenile and even larger *Youngina capensis* specimens (Gow, 1975). Therefore, several anatomical features of the postcranial skeleton cannot be attributed to ontogeny as previously suggested by Smith and Evans (1996).

The skull is clearly that of a neodiapsid judging by ventrolateral process of the parietal and is similar to that of *Youngina capensis*, however, there are several critical differences (see Discussion below). The skull is small, roughly half that of the known specimens of *Youngina capensis*, lacks dermal sculpturing, and has proportionately large orbits. The antorbital region of the skull is transversely broader than it is dorsoventrally tall and begins to narrow anteriorly towards a short and broad rostrum. This rostrum, measuring from the anterior tip of the skull to the anterior margin of the orbit, is longer than the posterior portion of the skull starting from the posterior margin of the orbit to the posterior end of the skull. The posterior margin of the skull is concave, displaying a medial embayment most prominent at the midline in dorsal view and lacks sagittal expansion. A large upper temporal fenestra and a larger lower temporal fenestra lacking a ventral margin define the rather gracile temporal architecture. Several wide denticle fields and a large suborbital fenestra and internal naris define the palate, while the braincase and mandible are broadly similar to that of *Youngina capensis* and other neodiapsid reptiles, with some exceptions (see Description below).

**Fig. 5:**
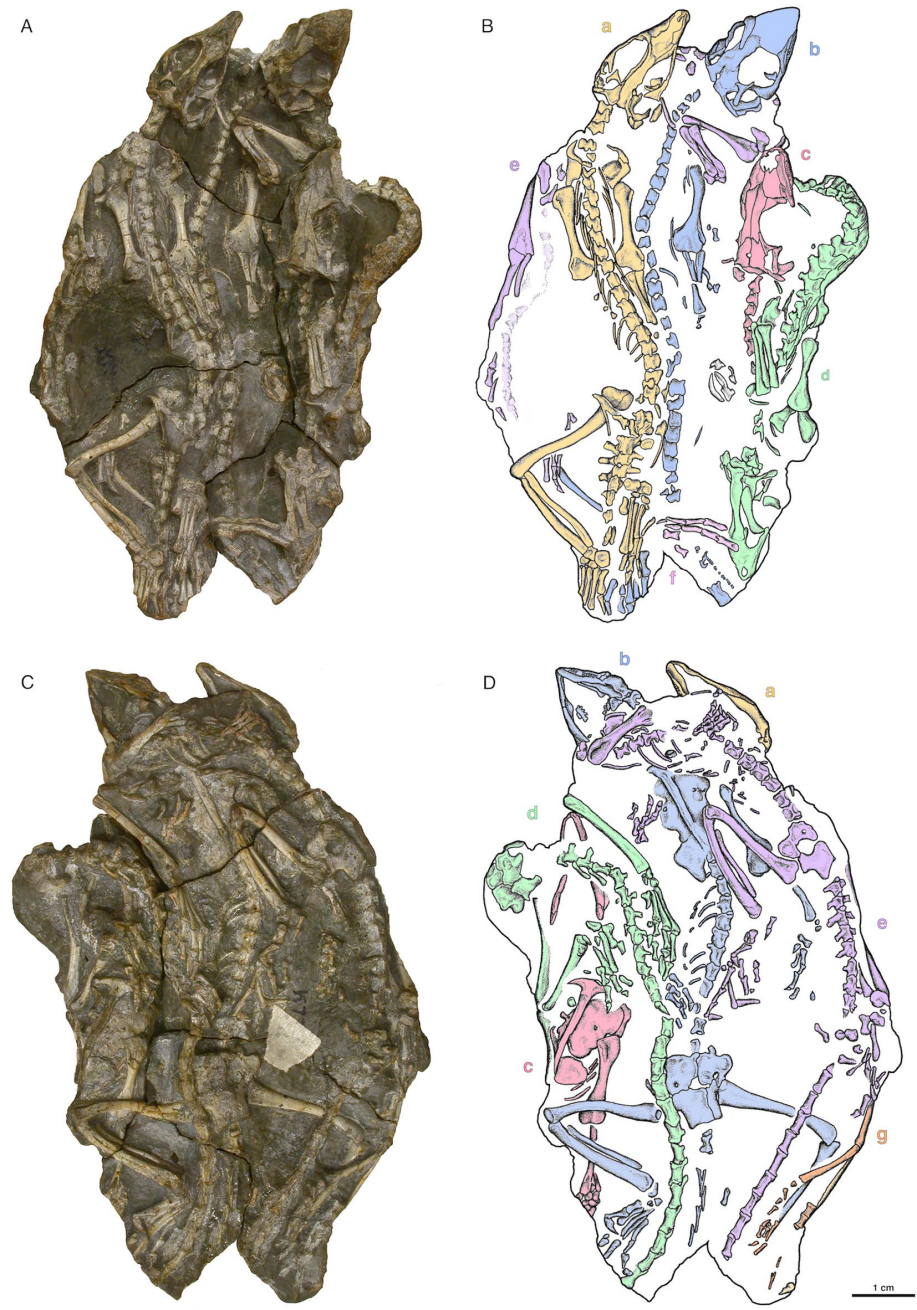
*Akkedops bremneri* aggregation SAM-PK-K7710 photographs and illustrations. **A** & **B** dorsal view. **C** & **D** ventral view. Each individual is colored and labeled as follows; SAM-PK-K7710a is denoted in yellow, SAM-PK-K7710b is denoted in blue, SAM-PK-K7710c is denoted in red, SAM-PK-K7710d is denoted in green, SAM-PK-K7710e is denoted in purple, SAM-PK-K7710f is denoted in pink, and SAM-PK-K7710g is denoted in orange

Nearly the complete postcranial anatomy of *Akkedops bremneri* is collectively represented by all individuals of SAM-PK-K7710 with the exception of some caudal vertebrae (Fig. 5). The postcranial skeleton is rather gracile and lizard-like with long slender limbs and a slender, long tail. The forelimbs are shorter than the hindlimbs and all presacral vertebrae have a transversely broader neural arch with respect to the centrum and have distinct double costal facets suggesting articulation of double headed costal ribs. Overall, the postcranial skeleton is most typical of terrestrial late Permian neodiapsids like *Saurosternon bainii* (Carroll, 1975) and *Youngina capensis* (Gow, 1975), but also the early Triassic lepidosauromorph *Palaeagama vielhauri* (Carroll, 1975). Given that SAM-PK-K7710 was previously described to some extent and includes measurements of the skeletons (Smith & Evans, 1996), we provide an additional comparative description that highlights new and otherwise neglected anatomies.

## Description

### Skull roof

The premaxilla is poorly preserved among all referred specimens, however, it is partially represented in the holotype SAM-PK-K6205 (Figs. 1 and 2). It is a paired bone forming the broad anterior-most extent of the rostrum and the anterior margin of the external naris. It sutures to the corresponding premaxilla medially and to the maxilla, nasal, and septomaxilla posteriorly. The dorsal process is subvertical at the base but begins to extend posterodorsally to the posteromedial margin of the external nares, for which the supranarial process also contributes to the separation of the external nares. A subnarial process is not visible, but it likely would have been short and end well anterior to the posterior margin of the external naris contrary to *Youngina capensis* (Gow, 1975). The homodont dentition is consistent with that of the maxilla in being similarly small, peg-like, and non-serrated with only slight recurvature at the crown apex.

Careful examination of the maxilla indicates that it is a low elongated bone (Figs. 1, 2, 3, 4). The maxilla sutures to the nasal and lacrimal dorsally, the premaxilla anteriorly, the jugal posteriorly, and forms the posterolateral margin of the external nares. It does not contribute to the orbital margin, nor does it contact the prefrontal anterior to the lacrimal and it lacks an antorbital fossa. The dorsal process reaches its maximum dorsoventral height immediately posterior to the external naris rather than centrally between the orbit and external naris as in *Youngina capensis* (Gow, 1975). The external naris is positioned marginally near the anterior most portion of the skull and its posterior margins are not posterodorsally expanded, to accomodate a rather wide and elongated external naris. A single row of supralabial foramina also extends along the lateral surface of the maxilla just above and parallel to the tooth row (Fig. 2). The dentition of the maxilla is also homodont, consisting of a single row of at least twenty-six relatively small, peg-like, non-serrated teeth with only very slight recurvature nearest the crown apex, which is less dramatic than those of *Youngina capensis* (Gow, 1975).

The lacrimal is a subtriangular bone contributing to the lateral surface of the rostrum directly anterior to the orbit and does not reach the posterior margin of the external nares (Figs. 1, 2, 3A–D, 4). It sutures to the nasal anterodorsally, the maxilla ventrolaterally, and the prefrontal and jugal posteriorly to exclude the maxilla from the orbital margin. It extends rather far anteriorly and tapers posteriorly to a thin sliver that makes a minor contribution to the orbital margin (Fig. 2). This contribution to the orbital margin is less than ¼ the orbital height, which is comparatively less than those of "younginiform" reptiles like *Youngina capensis* (Gow, 1975) and *Acerosodontosaurus piveteaui* (Bickelmann et al., 2009).

The nasal is poorly preserved in all referred *Akkedops bremneri* specimens except SAM-PK-K6205, which clearly demonstrates that it is subrectangular and transversely broad to form most of the dorsal and dorsolateral surface of the rostrum (Figs. 1, 2, 3 and 4E–G). It sutures to the premaxilla anteriorly, the maxilla laterally, the frontal, lacrimal, and prefrontal posteriorly, and forms part of the posteromedial margin of the external nares. The nasal is also half the anteroposterior length of the frontal (Fig. 1), which is proportionately shorter than many other stem saurians like *Youngina capensis* (Gow, 1975), and is more comparable to that of *Claudiosaurus germaini* (Carroll, 1981).

The prefrontal is a dorsoventrally tall, crescent-shaped bone forming nearly the entire anterior margin of the orbit and has an anterior sheet-like extension contributing to the posterolateral surface of the rostrum (Figs. 1, 2, 3, 4C–H). It sutures ventrally to the lacrimal and dorsally to the nasal and frontal. The ventral process is robust like that of *Lanthanolania ivakhnenkoi* (Modesto & Reisz, 2002) and makes a substantial contribution to the anterior margin of the orbit and would not contact the palatine.

The frontal is an unfused and paired bone that is well-represented in all referred skulls (Figs. 1, 2, 3, 4). It is transversely narrow and anteroposteriorly elongated, extending from the level of the anterior margin of the pineal foramen to the posterior nasal and contributes greatly to the dorsal margin of the orbit. It sutures to the posterior nasal via a broad imbricating suture, to the prefrontal and postfrontal via a tongue in groove suture, and to the parietal via a long and narrow U-shaped suture like that of *Youngina capensis* (Gow, 1975).

The parietal is a broad, paired bone forming much of the posterodorsal skull table (Figs. 1, 2, 3, 4, S1). It sutures to the corresponding parietal along the midline, the frontal anteriorly, the postfrontal anterolaterally, and to the supratemporal posterolaterally. The pineal foramen is bound by both parietals midway along the midline and it is less than ¼ of the interparietal length, which is rather small compared to *Youngina capensis* and all other “younginiform” reptiles (Gow, 1975). The ventrolateral process of the parietal is dorsoventrally narrow and extends slightly ventrally from the lateral margin of the parietal to form the dorsal margin of the upper temporal fenestra. This flange is typical of neodiapsids and is similar to that of *Claudiosaurus germaini* (Carroll, 1981), *Youngina capensis* (Gow, 1975), and the lepidosauromorph saurian *Pamelina polonika* (Evans, 2009). The posterolateral wings of the parietals are oriented posterolaterally at an angle of about 45**°** from midline to accentuate the concave posterior margin of the skull in dorsal view. They are also dorsoventrally shorter than the supraoccipital. The parietals also lack sculpturing or ridging on the dorsal surface. The smooth, slopping occipital shelf of the parietal indicates the absence of the postparietal and tabular. No evidence for a postparietal or tabular is evident in any referred *Akkedops bremneri* specimens, including the nearly completely represented individuals of SAM-PK-K7710.

The supratemporal is a small sliver-like bone of the posterolateral edge of the skull roof (Figs. 1, 2, 3, 4). It has a slender anteromedial process that dorsally sutures to the parietal and a broader posterolateral wing extending ventrolaterally suturing dorsally to the postorbital and squamosal. Rather unique to *Akkedops bremneri* is the distinct lateral flange of the posterolateral wing that sutures to the postorbital and contributes to the posterodorsal margin of the upper temporal fenestra. A suture between the posterior process of the postorbital and the supratemporal is not observed in any other Paleozoic diapsid. Although not in the same manner, such a contact is known in some synapsids like *Archaeovenator hamiltonensis* (Reisz & Dilkes, 2003) and *Varanops brevirostris* (Champione & Reisz, 2010), as well as some parareptiles like *Milleretta rubidgei* (Gow, 1972).

The postfrontal is an asymmetric T-shaped bone contributing to the upper temporal fenestra (Figs. 1, 2, 3, 4C-G). This is common among “younginiform” reptiles (Bickelmann et al., 2009; Carroll, 1981; Currie, 1981; Gow, 1975), but also the lepidosauromorph *Sophineta cracoviensis* (Evans & Borsuk-Bialynicka, 2009). This triradiate bone is primarily anteroposteriorly elongate, wherein the anterior and ventral processes form the posterodorsal margin of the orbit and a much smaller posterior process forms the anterodorsal margin of the upper temporal fenestra. It also sutures ventrolaterally to the postorbital, medially to the frontal and parietal, and is approximately less than half the size of the postorbital.

The postorbital is a prominent triradiate bone of the temporal region of the skull (Figs. 1, 2, 3, 4, 5, S1). It contributes to the posterior orbital margin, the anterior margins of both temporal fenestrae, and sutures to the jugal posteriroly, postfrontal anteriorly, squamosal posterolaterally, and supratemporal posteriorly. The angle between the ventral and posterior processes is approximately 90° and the ventral and dorsal processes display slight lateral thickening as part of the posterior margin of the orbit. The dorsal process is the shortest process of the postorbital and sutures posteriorly to the postfrontal to contribute to the anteroventral margin of the upper temporal fenestra. The posterior process is rather elongated, extending nearly the entire length of the temporal region and is rather narrow, measuring less than 20% of the dorsoventral height of the temporal region. A distinct recess of the lateral surface of the posterodorsal portion of the ventral process is also visible and is most pronounced at the junction with the posterior process. The ventral process also contributes to the anterodorsal margin of the lower temporal fenestra.

The jugal is an elongated triradiate bone composing the posteroventral margin of the orbit and anterior margin of the lower temporal fenestra. It sutures to the lacrimal, prefrontal, and maxilla anteriorly and the postorbital posteriorly (Figs. 1, 2, 3, 4A–D, H, S1). The anterior process is the longest process of the jugal and does not extend to the level of the anterior orbital margin, unlike that of *Youngina capensis* (Gow, 1975). The subtemporal process is narrow, greatly reduced, and ends freely, indicating the lack of a ventral margin of the lower temporal fenestra. This temporal architecture differs greatly from that of *Youngina capensis* (Gow, 1975) and *Acerosodontosaurus piveteaui* (Bickelmann et al., 2009) who both have a well-defined ventral margin of the lower temporal fenestra. This is, however, most similar to that of the stem saurian *Lanthanolania ivakhnenkoi* (Modesto & Reisz, 2002), *Claudiosaurus germaini* (Carroll, 1981), and lepidosauromorphs including *Paliguana whitei* (Ford et al., 2021). The dorsal process is thickened slightly laterally, which complements that of the postorbital to thicken the posterior margin of the orbit. The lateral surface of the jugal is smooth and there is no indication of tuberous striations.

The quadratojugal is greatly reduced to a slender splint-like bone suturing only to the quadrate below the ventral edge of the squamosal and is only visible in the μCT rendering of SAM-PK-K6205 (Fig. S1). A heavily reduced quadratojugal like this differs dramatically from that of *Youngina capensis* and *Acerosodontosaurus piveteaui* (Bickelmann et al., 2009), and is most similar to *Claudiosaurus germaini* (Carroll, 1981) and is typical of many early diverging saurians (Ezcurra et al., 2014). The dorsal process is also short and limited to the posteroventral corner of the skull.

The squamosal is slender and elongated with minimal dorsal expansion (Figs. 1, 2, 3, 4A–D, H, S1). It resembles most closely that of *Acerosodontosaurus piveteaui* (Bickelmann et al., 2009), *Claudiosaurus germaini* (Carroll, 1981), and the lepidosauromorph *Paliguana whitei* (Ford et al., 2021). It sutures to the parietal and supratemporal dorsomedially, the quadrate and quadratojugal ventrally, and forms the posterolateral margin of the lower temporal fenestra. A squamosal-parietal contact may exist judging by the elongated and slender morphology of the squamosal; however, it would be minimal and be covered laterally by the supratemporal. The lateral flange of the squamosal is anteroposteriorly narrow and braces the lateral margin of the quadrate. There is also no indication of posterior lamina, thereby exposing the posterior portion of the quadrate occipitally like in many saurians.

The quadrate is a laterally broad, saddle-shaped bone that articulates the skull roof to the mandible. It has several distinctive morphologies best shown in the μCT rendering of the holotype skull SAM-PK-K6205 (Figs. 1, 2, 3, 4E, F, S1, S2). It sutures to the quadratojugal anterolaterally, squamosal laterally, and the parietal dorsally to form the ventral portion of the posterolateral edge of the skull. The quadrate is laterally concave, although it does not appear to have a distinct lateral conch or notch, as is also observed in the lepidosauromorph *Paliguana whitei* (Ford et al., 2021). The μCT data reveals a moderately pronounced ventrodorsally trending lateral flange extending the dorsoventral height of the quadrate, which is consistent with a tympanic crest and is a typical saurian feature (Ezcurra et al., 2014). The posterior pillar is slender and the dorsal condyle tapers slightly to suture to the squamosal, although a peculiar moderate flange/shelf also appears to extend posteriorly from the posterior pillar. A unique medial ridge directly above the anteroventral condyle likely braced the convex lateral surface of the quadrate ramus of the pterygoid, which appears to be a rather unique feature of *Akkedops bremneri*. The ventral condyle has an anteriorly concave crescent shape, and the posterior surface is heavily emarginated, a feature also typical of early diverging saurians (Ezcurra et al., 2014). The quadrate would have a nearly vertical orientation in lateral view and indicates that the jaw would articulate approximately level with the occiput and the alveolar margin of the maxilla. The broad anteriorly convex surface, posterior emargination, and posterolateral pronunciation of the posterior pillar are also somewhat similar to that of the lepidosauromorph *Pamelina polonika* (Evans, 2009).

### Palate

The vomer is currently only visible in the μCT rendering of the holotype SAM-PK-K6205 (Fig. S3). It is an elongated, plate-like, and triangular-shaped bone of the palate with a dentulous ventral surface. The lateral surface of the vomer forms the medial margin of the internal naris and is sutured to the premaxilla anteriorly, the corresponding vomer medially, the palatine posteriorly, and the pterygoid posteromedially. The anteroposterior length of the vomer is proportionately shorter than that of *Youngina capensis* (Gow, 1975), which is consistent with the proportionately shorter rostrum of *Akkedops bremneri*. The anterior terminus of the vomer is also a single process rather than being bifurcated like in *Youngina capensis* (Gow, 1975; Hunt et al., 2023). The ventral surface exhibits two raised densely populated denticle fields that are roughly three denticles wide and each denticle is considerably smaller than those of the marginal dentition. The medially positioned denticle field extends from the pterygoid along the midline. The laterally positioned denticle field appears to extend from the palatine and trends just medial to the internal margin of the internal naris. This differs from the denticle fields of *Youngina capensis* which are typically single denticle rows (Gow, 1975; Hunt et al., 2023).

The palatine is best exposed in the holotype μCT rendering of SAM-PK-K6205 and the dorsal surface is visible in BP/1/2614 (Fig. S3). It is a plate-like, dentulous bone suturing anteriorly to the vomer, medially to the corresponding palatine, and laterally to the maxilla and pterygoid. The ventral surface of the palatine contains denticles about twice the size of those on the pterygoid and vomer. A single, densely populated, and raised denticle field ranging from three to five denticles in width, trends anterolaterally toward the vomer, again, differing dramatically from the reduced denticle fields of *Youngina capensis* (Hunt et al., 2023). The lateral margin of the vomer forms the posteromedial and posterior margin of the internal naris, as well as a curved anteromedial margin of the suborbital fenestra, which are separated from one another by a well-defined maxillary ramus. The suborbital fenestra is also relatively wide and elongated compared to *Youngina capensis*, as is the internal naris (Gow, 1975; Hunt et al., 2023).

The pterygoid is clearly visible in the holotype SAM-PK-K6205 and the complete anatomy is shown in the corresponding μCT rendering (Figs. 1, S3). It is an elongated, irregularly shaped, plate-like bone suturing to the palatine anterolaterally, the corresponding pterygoid medially along its anterior-most margins, the epipterygoid dorsomedially, and the ectopterygoid laterally. The ventral surface is gently convex and has several distinct denticle fields. There are two raised denticle fields of slightly recurved denticles that are considerably smaller than those of the marginal dentition. The medial denticle field is approximately three denticles wide, running the anteroposterior extent of the pterygoid along the midline and is most prominently raised to merge with the medial denticle field of the vomer. A single raised denticle field roughly four denticles wide extends anterolaterally to merge with that of the palatine, which differs from the two distinctly separated, single denticle wide lateral denticle fields present in *Youngina capensis* (Hunt et al., 2023). A wide denticle field equivalent in size to those elsewhere on the pterygoid also populates the ventral surface of the transverse flange of the pterygoid, which differs from the single row of proportionately larger denticles observed in *Youngina capensis* (Hunt et al., 2023). The transverse flange of the pterygoid is directed laterally to form an obtuse angle with the palatal process in ventral view and the lateral margin appears rectangular. The quadrate ramus of the pterygoid exhibits an arcuate flange that lacks dentition and is somewhat medially concave, similar to that of *Youngina capensis* (Gow, 1975; Hunt et al., 2023). It would likely have articulated laterally within the medial conch ventral to the medial shelf of the quadrate.

The epipterygoid is only visible in the μCT rendering of the holotype SAM-PK-K6205 (Fig. S3). It has a narrow dorsal columella and a heavily spatulated ventral process that articulates with the dorsal recess of the quadrate ramus of the pterygoid. The epipterygoid also clearly does not contribute to the basicranial articulation.

The ectopterygoid is visible in BP/1/2614 (Fig. 3). It is a somewhat rectangular, plate-like bone that sutures with the pterygoid, palatine, and jugal to form the posterior and posteromedial margins of the suborbital fenestra and the anterior margin of the subtemporal fenestra. The ectopterygoid is rather large in *Akkedops bremneri*, with a short robust jugal ramus and a gently concave emargination of its anterolateral margin to form a wide posterior margin of the suborbital fenestra. It also does not appear likely that it reached the posterolateral corner of the transverse flange of the pterygoid.

### Braincase

The supraoccipital is visible in the μCT rendering of the holotype SAM-PK-K6205 and BP/1/2614 (Figs. 1, 2, 3, S4). It is a symmetrical bone forming the posterodorsal portion of the braincase, the dorsal margin of the foramen magnum, and a minor portion of the posterior semicircular canal. It has the typical broad configuration, wherein its posterior margin is gradually emarginated toward the midline. It also sutures anterolaterally to the prootic, laterally to the opisthotic, posteriorly to the exoccipitals, and dorsally to the parietals. The posterior surface appears smooth, lacking a midline crest. A depression on the posterior surface near the suture with the prootic and opisthotic likely indicates where the parietal would suture. There also appears to be a large groove midway along the lateral margin of the supraoccipital that is complemented by a similar invagination of the dorsomedial margin of the opisthotic. There is also no lateral expansion for the dorsal margin of a posttemporal fenestra.

The exoccipitals are highlighted well by the μCT rendering of SAM-PK-K6205 (Figs. 1, 2, 3, S4). They form the lateral margins of the foramen magnum and are fused with the basioccipital but not to the opisthotics. The dorsomedial processes of the exoccipitals are more robust and distally expanded than those of *Youngina capensis* (Gardner et al., 2010) and do not meet at the midline, thereby allowing contribution of the supraoccipital to the foramen magnum. The foramina marking the exit of the hypoglossal nerve (CN XII) is also clearly visible on each exoccipital in occipital view (Fig. S4A, B).

The basioccipital is well-defined in both the SAM-PK-K6205 μCT rendering and BP/1/2614 (Figs. 1, 2, 3, S4). It contributes to the posteroventral portion of the braincase and forms the ventral margin of the foramen magnum. It sutures laterally to the opisthotics, anteriorly to the posterior parasphenoid, and is completely fused to the exoccipitals. The basioccipital condyle displays a well-defined elliptical notochordal depression that comprises most of the posterior surface. There are also well-developed and ossified basal tubera extending ventral to the occipital condyle and would have contacted the alar wings of the parasphenoid.

The parabasisphenoid is best represented in the μCT rendering of SAM-PK-K6205 (Figs. 1, 2, 3, S4) but is also partially visible in BP/1/2614. The parasphenoid and basisphenoid are not only fused and are ventrodorsally compressed, the latter reflects lithostatic compression. The parasphenoid sutures to the pterygoids ventrally and to the prootics dorsally. The cultriform process is a narrow and elongated anterior extension of the parasphenoid that is oriented horizontal to the long axis, which appears proportionately shorter than that of *Youngina capensis* (Gardner et al., 2010; Hunt et al., 2023). The base of the cultriform process is typically widened and has few isolated denticles on its ventral surface that appear to trend anteroposteriorly (Fig. 1C, D). The ventral surface of the parasphenoid is depressed as is typical of many early diapsids and lacks dentition unlike *Youngina capensis* (Hunt et al., 2023). It appears that the posterior most portion of the basisphenoid between the alar wings is unossified and the surface is smooth. The short widely spaced basipterygoid processes are anterolaterally oriented to articulate with the basicranial recess of the pterygoid and are level with the transverse flange of the pterygoid. A vidian sulcus is also observable as a depression hugging the lateral edge of the crista ventrolateralis like that of *Youngina capensis* (Hunt et al., 2023). There is also a foramen for entry of the internal carotid arteries on the ventral surface medial to the basipterygoid processes. The Parasphenoid crests are prominent ventrolateral extensions that bound the ventromedial floor of the vidian canal and the pronounced cristae ventrolaterales accentuate the median concavity between them referred to as the pharyngeal recess.

The opisthotic is represented in the holotype SAM-PK-K6205 and clearly shown in the corresponding CT rendering but is also partially visible in BP/1/2614 (Figs. 1, 2, 3, S4). It sutures to the prootic, supraoccipital, and exoccipital to enclose much of the vestibular system and form the posterolateral wall of the braincase. The large ventral ramus of the opisthotic forms an anteroposterioly narrow plate and contributes heavily to the posterolateral margin of the braincase. The paraoccipital processes are not well-represented but appear slender and taper distally, however, it is unclear if they contacted the quadrate.

The prootic is best represented in the μCT rendering of the holotype SAM-PK-K6205 (Figs. 1, 2, S4). It has a well-developed inferior rostral process framing the anterior margin of the trigeminal foramen, which is also observed in *Youngina capensis* as a small process (Gardner et al., 2010) and is common in archosauromorphs like *Euparkeria capensis* (Sookias et al., 2020). The prominent anterolateral ridge containing the rostral portion of the horizontal semicircular canals of the inner ear are also like that of *Youngina capensis* (Gardner et al., 2010).

The stapes is preserved in the holotype SAM-PK-K6205 (Figs. 1, S4). It is very similar to that of *Youngina capensis* in having a small footplate and a relatively thick rod-like shaft with a large stapedial foramen for the stapedial artery (Gardener et al., 2010). However, it remains unclear if there is a dorsal process.

### Mandible

The dentary is well-represented in the holotype SAM-PK-K6205 and is the largest mandibular bone (Figs. 1, 2, 3, 4H). It comprises most of the anterolateral and much of the ventral surfaces of the mandible. It sutures to the corresponding dentary anteromedially at the mandibular symphasis and to the splenial, prearticular, and angular medially. Its posterior process is sutured to the surangular dorsally and to the angular ventrolaterally, while the coronoid is sutured atop its posterodorsal surface. The mandibular symphysis is formed only by the dentaries and the anteroventral surface displays clusters of small foramina. Each dentary has a single row of small, peg-like, non-serrated teeth that have a modest recurvature nearest the crown, just like those of the maxilla and premaxilla.

The angular is well-represented in nearly all referred skulls (Figs. 1, 2, 3, 4H). It appears U-shaped in cross-section to form most of the posteroventral and much of the posterolateral surfaces of the mandible, although it contributes comparatively less to the lateral surface than that of *Youngina capensis* (Hunt et al., 2023). It is sutured anterolaterally to the dentary, anteromedially to the splenial, medially to the prearticular, laterodorsally to the surangular, and posteriorly to the articular.

The surangular is most easily visible in the holotype SAM-PK-K6205 and BP/1/2614 (Figs. 1, 2, 3). It forms the dorsolateral surface of the posterior half of the mandible, more so than the angular. This is proportionately more than that of *Youngina capensis* (Hunt et al., 2023) and is more similar to *Claudiosaurus germaini* (Carroll, 1981). It sutures anteriorly to the dentary and coronoid, ventrally to the angular and prearticular, and posteriorly to the articular. The anatomy of the posterior shelf is not visible in any specimen. There is also no anterior lateral foramen near the suture with the dentary and the surangular extends anteriorly past the coronoid eminence.

The splenial is visible in the holotype SAM-PK-K6205 and BP/1/2614 (Figs. 1, 2, 3). It is a slender blade-like bone forming the anteromedial surface of the mandible and does not appear to contribute to the mandibular symphysis. It sutures to the dentary laterally along its anteroposterior length and to the prearticular, angular, and coronoid posterolaterally.

The coronoid is not separated into anterior and posterior portions and is exposed in the holotype SAM-PK-K6205 and BP/1/2614 (Figs. 1, 3). It is a small edentulous bone positioned atop the dorsal surface of the posterior process of the dentary and extends to contact the surangular, splenial, and possibly the prearticular. The knob-like dorsal process of the coronoid is well-developed but is not tall.

The prearticular is visible in the holotype SAM-PK-K6205 and BP/1/2614 (Figs. 1, 3). It is a slender and elongated bone forming a long section of the posteromedial surface of the mandible. It is posteriorly expanded to buttress the articular, while also suturing anteriorly to the dentary and splenial, ventrally to the angular, and dorsally possibly to the coronoid.

The articular is visible in the holotype SAM-PK-K6205 and BP/1/2614 (Figs. 1, 2, 3). It is a small crescent-shaped bone forming the posterior-most terminus of the mandible and sutures to the posterior end of the prearticular and surangular to articulate the mandible with the skull. The retroarticular process is formed solely by the articular and is dorsoventrally shallow. The dorsal margin is posteroventral to the quadrate articulation and the articular surface is anteroposteriorly downturned. It also does not extend far beyond the quadrate posteriorly, which is in strong contrast to many archosauromorphs.

### Axial skeleton

**Fig. 6:**
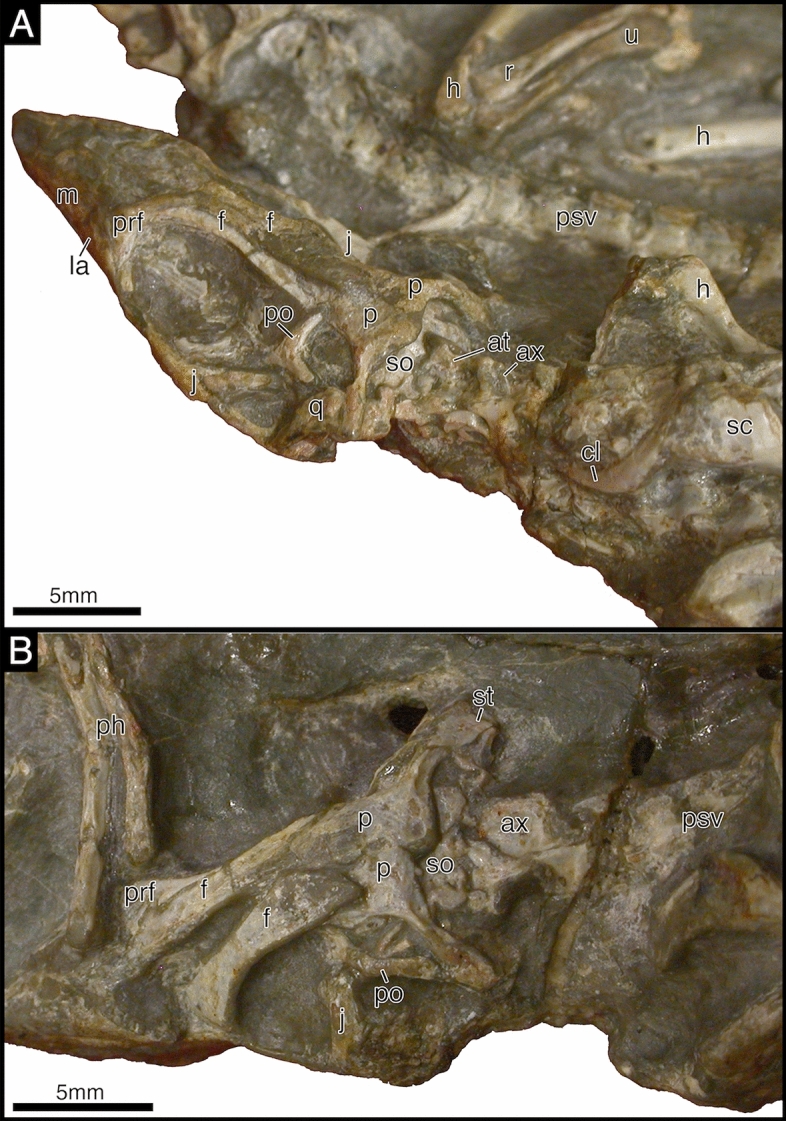
*Akkedops bremneri* SAM-PK-K7710a and SAM-PK-K7710d photographs of atlas axis complex. **A** SAM-PK-K7710a left posterolateral view. **B** SAM-PK-K7710d left dorsolateral view. *ax* axis, cl clavicle, *f* frontal, *h* humerus, *j* jugal, *la* lacrimal, *m* maxilla, *p* parietal, *pf* postfrontal, *ph* phalange, *po* postorbital, *prf* prefrontal, *psv* presacral vertebra, *q* quadrate, *r* radius, *sc* scapula, *so* supraoccipital, *st* supratemporal, *sq* squamosal, *u* ulna

The atlas axis complex is represented in SAM-PK-K7710a, BP/1/2614, and to a lesser extent in SAM-PK-K7710d (Fig. 3, 6) but much of the anatomy remains obscured. The spine of the axis is anteroposteriorly long and mediolaterally narrow. It is unclear if the axial intercentrum is separated from the atlantal centrum.

**Fig. 7:**
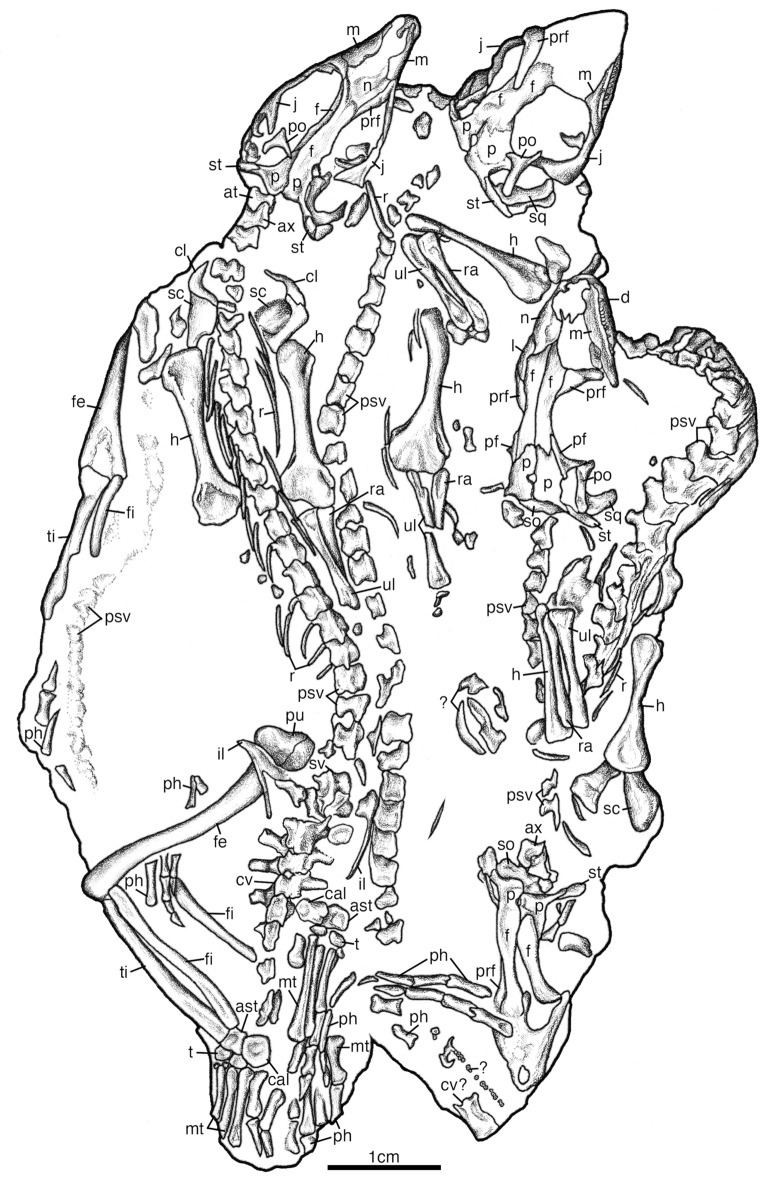
Illustration of *Akkedops bremneri* SAM-PK-K7710 dorsal view. *ast* astragulus, *ax* axis, *at* atlas, *cal* calcaneum, *cl* clavicle, *cv* caudal vertebra, *d* dentary, *f* frontal, *fe* femur, *fi* fibula, *h* humerus, *il* ilium, *j* jugal, *m* maxilla, *mt* metatarsal, *n* nasal, *l* lacrimal, *p* parietal, *pf* postfrontal, *ph* phalange, *po* postorbital, *psv* presacral vertebra, *pu* pubis, *prf* prefrontal, *r* rib, *ra* radius, *sc* scapulacoracoid, *so* supraoccipital, *sq* squamosal, *st* supratemporal, *sv* sacral vertebra, *t* tarsal, *ti* tibia, *ul* ulna, *?* unknown

**Fig. 8:**
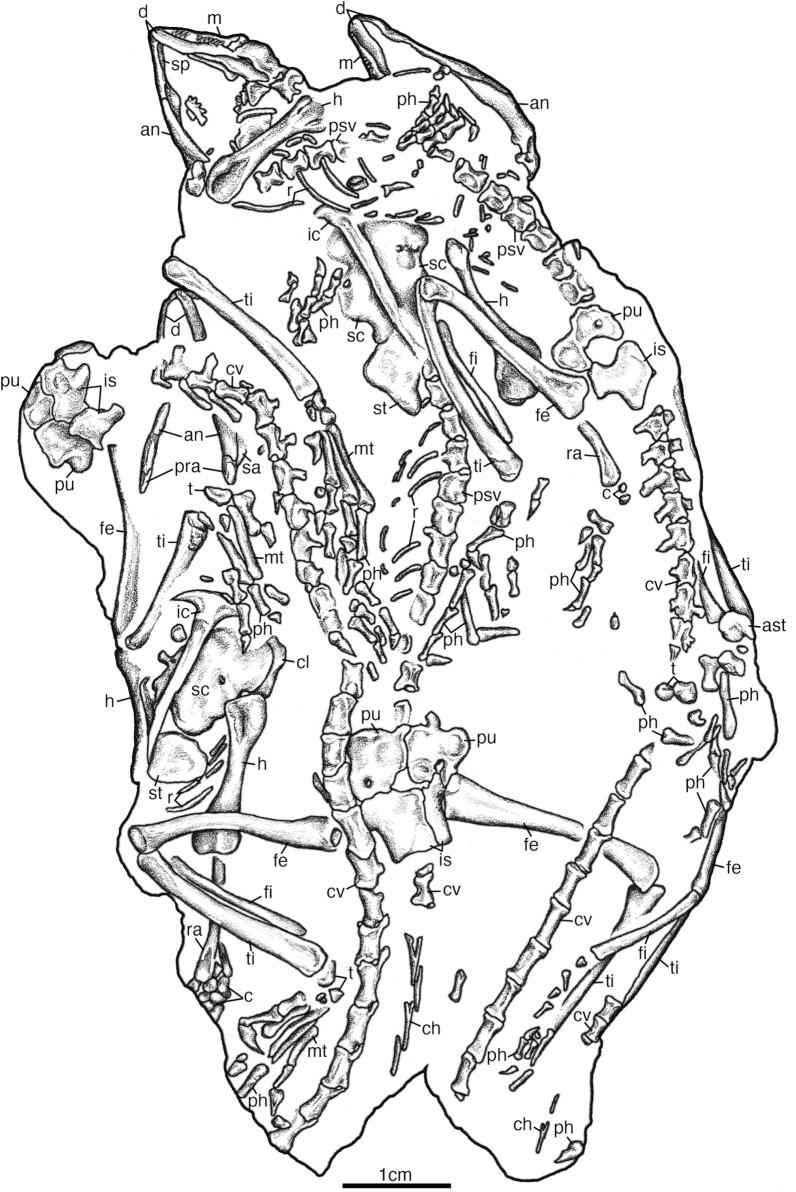
Illustration of *Akkedops bremneri* SAM-PK-K7710 ventral view. *an* angular, *ast* astragulus, *c* carpal, *cl* clavicle, *cv* caudal vertebra, *ch* chevron, *d* dentary, *fe* femur, *fi* fibula, *h* humerus, *ic* interclavicle, *is* ischium, *m* maxilla, *mt* metatarsal, *ph* phalange, *pra* prearticular, *psv* presacral vertebra, *pu* pubis, *r* rib, *ra* radius, *sa* surangular, *sc* scapulacoracoid, *sp* splenial, *st* sternal plate, *t* tarsal, *ti* tibia

**Fig. 9:**
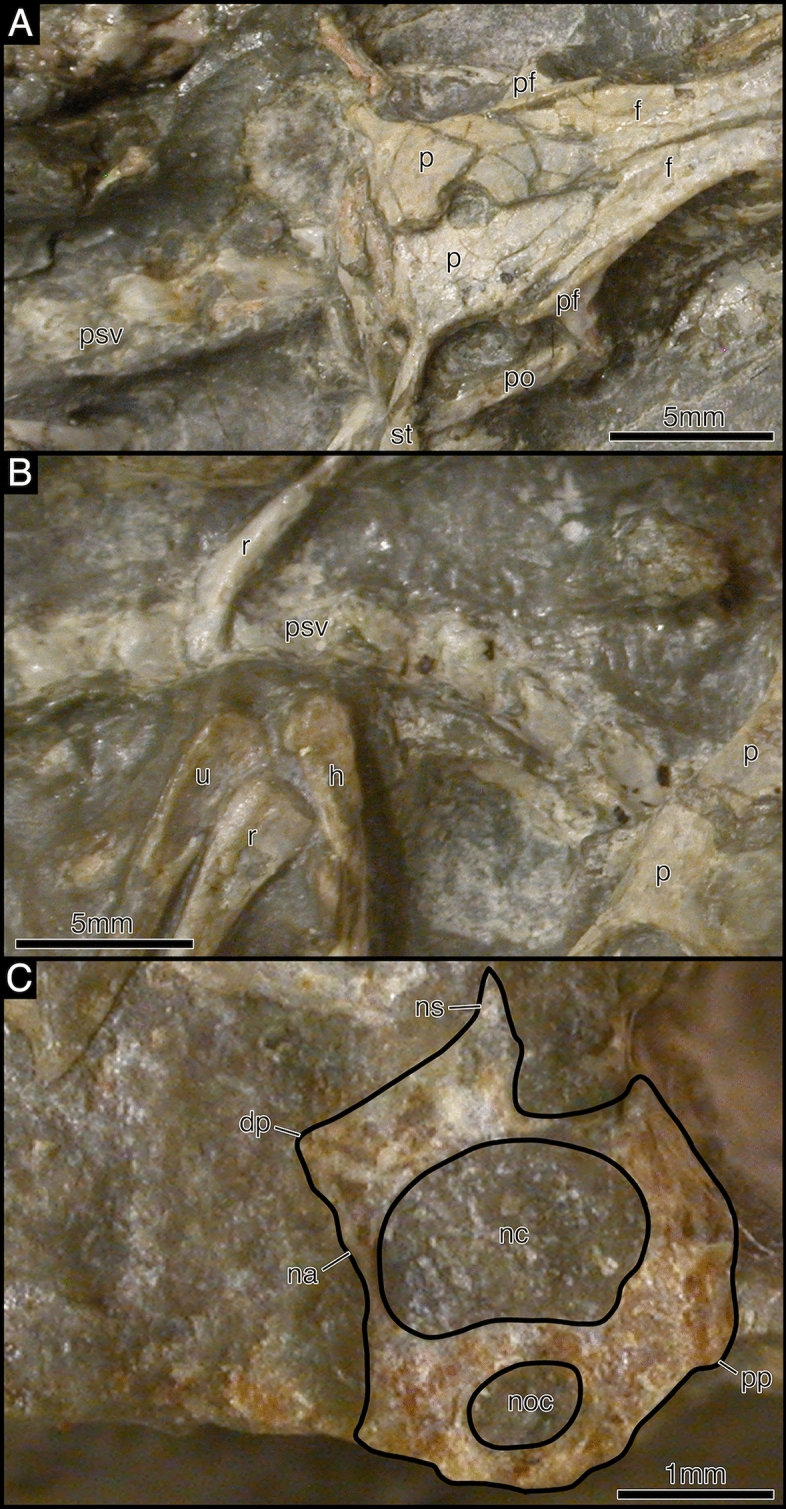
*Akkedops bremneri* SAM-PK-K7710 anterior presacral vertebrae. **A** SAM-PK-K7710c dorsal view. **B** SAM-PK-K7710b dorsal view. **C** SAM-PK-K7710a 3^rd^ presacral vertebra cross-section transverse view. *dp* diapophysis, *f* frontal, *h* humerus, *na* neural arch, *nc* neural canal, *noc* notochordal canal, *ns* neural spine, *p* parietal, *pf* postfrontal, *po* postorbital, *pp* parapophysis, *r* radius, *st* supratemporal, *tp* transverse process, *u* ulna

There is a total of twenty-nine presacral vertebrae. The anterior presacral vertebrae are exposed in SAM-PK-K7710a-c (Figs. 7, 8, 9). All of which are slightly more elongated than the posterior presacral vertebrae and have distinctly separated diapophyses and parapophyses, suggesting the articulation of double headed ribs like in *Claudiosaurus germaini* (Carroll, 1981) (Fig. 9). The anterior presacrals have notochordal amphicoelous centra that lack hypapophyses. They are also hourglass-shaped in ventral view with a low longitudinal median ridge and are broadly rectangular in lateral view. The articular surfaces of the prezygapophyses are oriented medially and are complemented by the laterally oriented articular surfaces of the postzygapophyses that also lack epipophyses. The neural spines are short, anteroposteriorly long, and subrectangular in lateral view with some lateral swelling at the base (Fig. 9a), while the anterior post-axial cervical vertebrae have a posterodorsally inclined anterior margin of the neural spine.

**Fig. 10:**
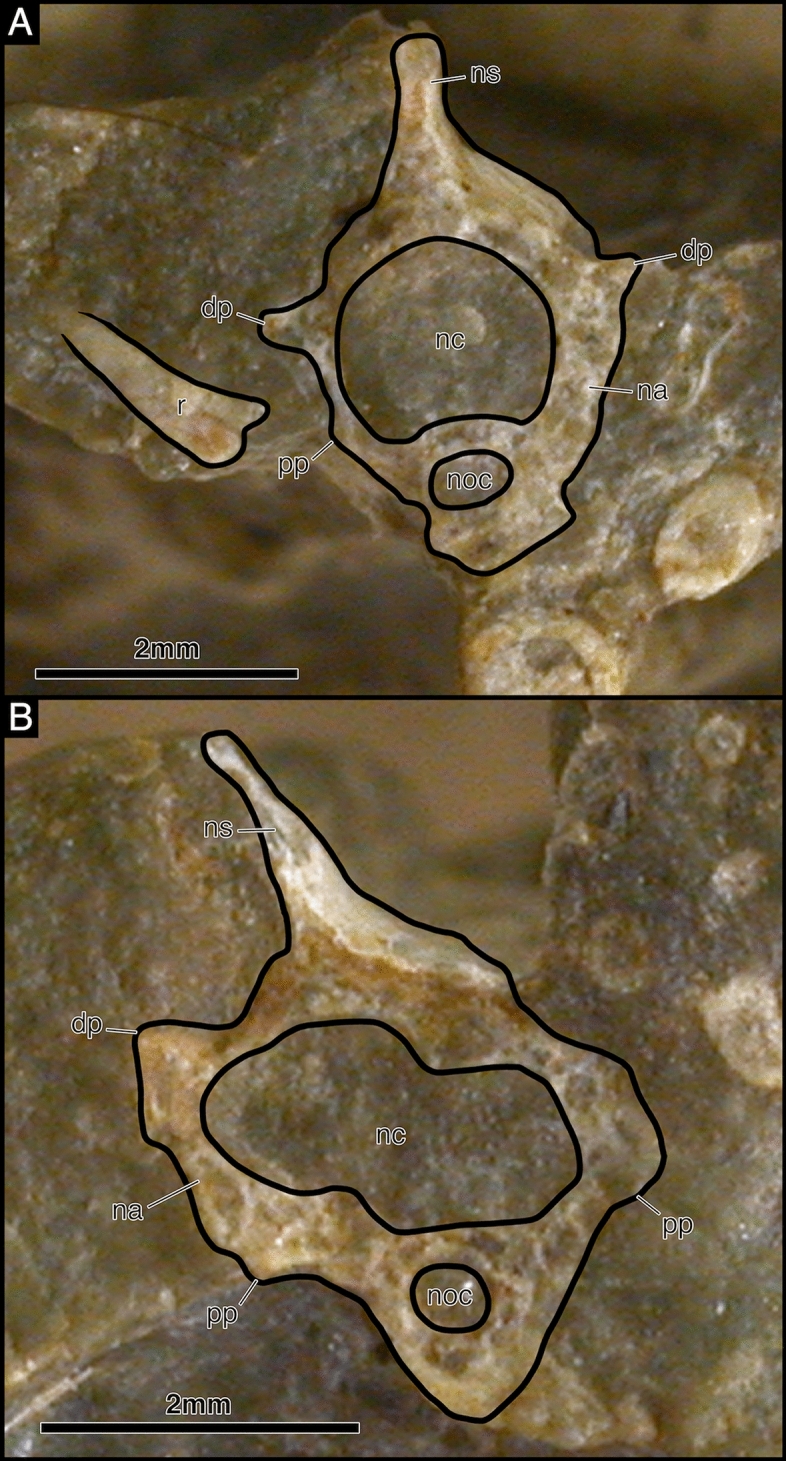
*Akkedops bremneri* SAM-PK-7710 K posterior presacral vertebrae in cross-section. **A** SAM-PK-K7710b 12th presacral vertebra transverse view. **B** SAM-PK-K7710b 23rd presacral vertebra transverse view. *dp* diapophysis, *nc* neural canal, *noc* notochordal canal, *ns* neural spine, *pp* parapophysis, *r* rib

The posterior presacral vertebrae are clearly visible in most SAM-PK-K7710 individuals (Figs. 7, 8, 10). These vertebrae remain notochordal and amphicoelous (Fig. 10), each of which also articulates with long, slender, and gently curved thoracic ribs. No proximal portion of any costal rib is visible, however, the occurrence of distinctly separated diapophyses and parapophyses suggests double headed costal ribs (Fig. 10). This differs from the largely single headed costal ribs of the posterior presacral vertebrae of many “younginiform” reptiles, however, is observed in *Claudiosaurus germaini* (Carroll, 1981), the weigeltisaurid *Coelurosauravus elivensis* (Buffa et al., 2022), and earlier diverging diapsids like *Araeoscelis gracilis* (Vaughn, 1955). The diapophyses are positioned lateral to the transverse processes and the parapophyses are located on the ventrolateral margin of the centrum. The ventral surface of each centrum displays a low longitudinal median ridge like that of *Palaeagama vielhauri* and is distinct from the more rounded condition seen in *Saurosternon bainii* (Carroll, 1975). Of the middle and posterior presacral vertebrae, the dorsoventral height of the pedicel is also much greater than the respective centra contrary to most “younginiform” reptiles. The neural arch is transversely broad and extends laterally beyond the lateral margin of the centrum unlike most “younginiform” reptiles and lacks a postzygapophyseal buttress. There is also a lack of dorsal excavations lateral to the base of the neural spine, which differs from many archosauromorph saurians (Ezcurra et al., 2014) but also Permian araeoscelidians (Reisz, 1981; Vaughn, 1955) and varanopid synapsids (Berman & Reisz, 1982; Campione & Reisz, 2010; Reisz & Dilkes, 2003). The neural spines are subrectangular in shape, being dorsoventrally shorter than they are anteroposteriorly long and each has a nearly vertical anterior margin. The neural spines also appear mediolaterally narrow, lack any texturing, and are not laterally swollen at their base.

The sacral vertebrae are best represented in the μCT rendering of SAM-PK-K7110e, which clearly shows two distinct sacral vertebrae (Fig. S5). They are also visible as fragmentary elements in SAM-PK-K7110a (Fig. 7). The sacral ribs of each vertebra are equally sized and distally expanded, however, the sacral rib of the second sacral vertebra is bifurcate with two nearly equal sized distal ends. This is similar to some archosauromorphs like *Prolacerta broomi* (Gow, 1975).

The caudal vertebrae are elongated and dorsoventrally short with low neural spines, as seen in SAM-PK-K7710d (Figs. 7, 8). The preserved portions of the tail are represented by at least twenty-six caudal vertebrae that are distinctly anteroposteriorly longer than the presacral vertebrae. Those currently observable also show little evidence of any successive decrease in their size throughout the caudal series, suggesting that the tail is rather long like that of *Saurosternon bainii* and *Palaeagama vielhauri* (Carroll, 1975). The transverse processes of the anterior-most five caudal vertebrae lack caudal ribs and are straight, extending perpendicular to the long axis of the vertebrae. The first caudal vertebra has the longest transverse processes, while the fifth caudal vertebra has the shortest, like that of *Saurosternon bainii* as well as *Yougnina capensis *(Carroll, 1975; Gow, 1975). This condition is not only observed in “younginiform” reptiles (Gow, 1975; Harris & Carroll, 1977; Carroll, 1981; Currie et al., 1981) and is most similar to *Saurosternon bainii* (Carroll, 1975), but also several late Permian and early Triassic saurians (Ezcurra et al., 2014; Ezcurra, 2016), as well as some Permian parareptiles like *Milleretta rudibgei* (Gow, 1972). The neural spine is not clearly visible in any individual, but they appear to have typical low neural spines. The chevrons are unfused to their centra and are Y-shaped in anteroposterior view and are straight and plate-like in lateral view (Fig. 7, 8). They also lack any anterior process and are of uniform width.

### Appendicular skeleton

The clavicle is visible in SAM-PK-K7710a and SAM-PK-K7710c (Figs. 6, 7, 8). It is a paired crescent-shaped bone of the anterior shoulder girdle suturing to the interclavicle and scapula. There is also no trace of a cleithrum preserved in any skeleton, but its presence cannot be ruled out.

**Fig. 11:**
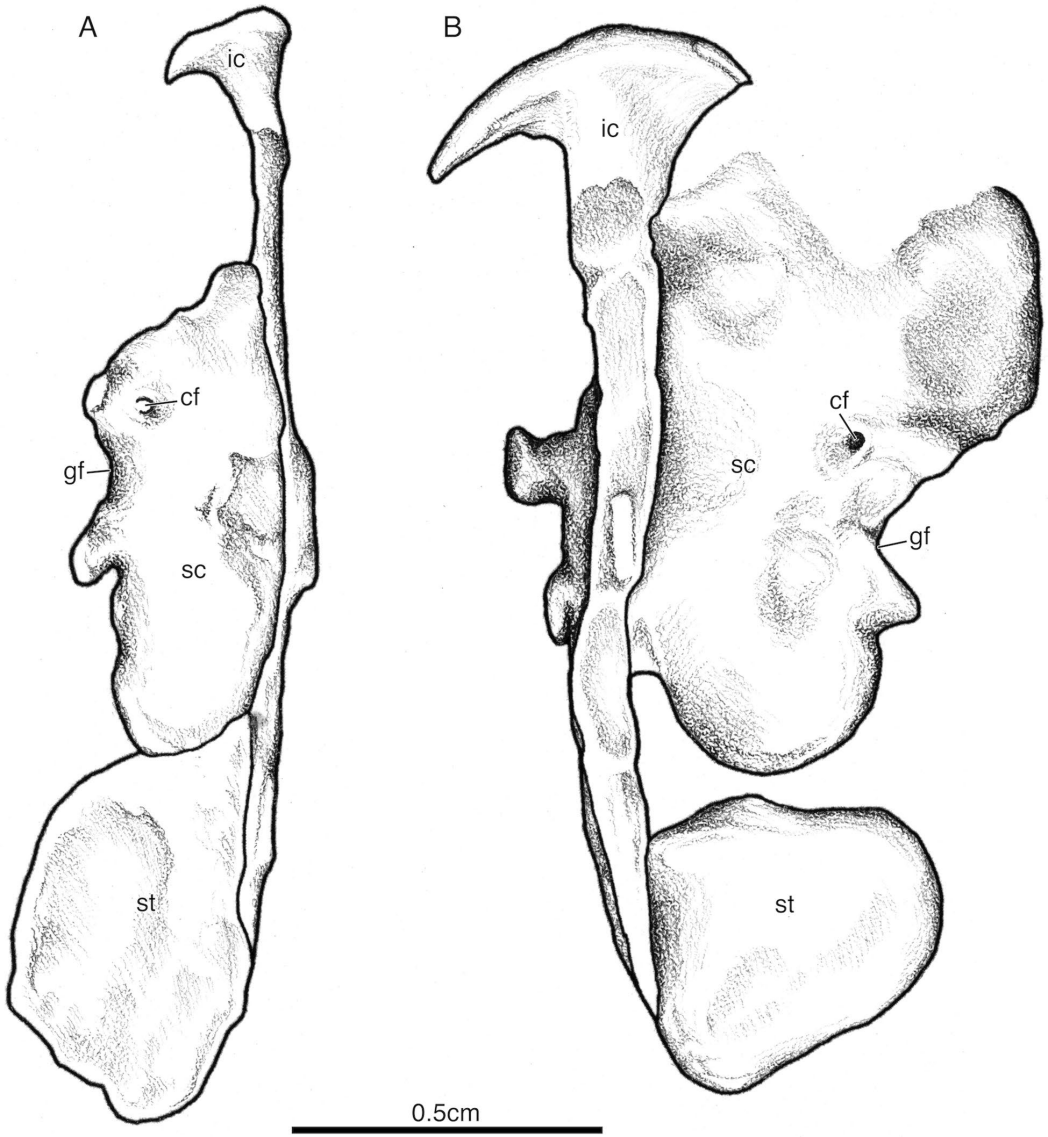
*Akkedops bremneri* shoulder girdle illustrations. **A** SAM-PK-K7710c ventral view. **B** SAM-PK-K7710b ventral view. *cf* coracoid foramen, *gf* glenoid fossa, *ic* interclavicle, *sc* scapulacoracoid, *st* sternal plate

The interclavicle is visible in individuals SAM-PK-K7710b-c (Figs. 8, 11). It is a prominent T-shaped bone of the ventral shoulder girdle positioned along the midline to underlie the clavicles and contact the scapulacoracoids and sternal plates. The anterior margin is smoothly convex lacking an anterior process and the lateral processes are long and with heads about twice as long as they are wide. The plate of the interclavicle is reduced to a narrow bar and the lateral processes are oriented gently posterolaterally as in *Saurosternon* bainii (Carroll, 1975). The posterior process is also rather anteroposteriorly long, especially relative to many saurians.

The scapulacoracoid is best exposed in SAM-PK-K7710b-c (Figs. 8, 11). It is a paired, plate-like bone lacking an obvious notch along its anterior margin, reflecting the scapula-coracoid suture just like in *Youngina capensis* (Gow, 1975). The glenoid fossa is oriented more laterally than posteriorly. The scapular blade is mostly dorsally inclined, rectangular in outline, and appears tall, although its exact height cannot be determined. There is also no evidence for a supraglenoid buttress or foramen unlike that of *Saurosternon bainii* (Carroll, 1975). The coronoid appears to have an L-shaped posterior border in lateral view due to its strong posterior expansion.

A left sternal plate is visible in SAM-PK-K7710c and a right sternal plate is visible in SAM-PK-K7710b, both remain articulated lateral to the stem of the interclavicle along half their medial anteroposterior length (Figs. 8, 11). They completely encompass the interclavicle stem laterally and are positioned directly along the posteromedial margin of the scapulacoracoid. This pair of unfused and ovoid-shaped sternal plates appears typical of stem saurians for which sternal ossifications are known (e.g., *Hovasaurus boulei* [Currie, 1981], *Tangasaurus mennelli* [Currie, 1982], *Kenyasaurus mariakaniensis* [Harris & Carroll, 1977], *Saurosternon bainii* [Carroll, 1975]).

The humerus is a slender bone of the forearm and is roughly 25% longer than the radius and ulna (Figs. 7, 8, S6). It has modestly expanded distal ends similar to *Palaeagama vielhauri* (Carroll, 1975) and there appears to be a torsion of approximately 45° between the proximal and distal ends like in all “younginiform” reptiles. In anterior view, the proximal end is roughly symmetrical in outline with a small central depression possibly indicating the presence of an unossified ball-shaped articulating surface. Perhaps this is representative of an epiphyseal ossification like that suggested for *Saurosternon bainii* and seen in modern lizards (Carroll, 1975), although its presence cannot be confirmed. The distal capitellum and trochlea are not well-developed and are distinct from the ectepicondyle and entepicondyle. The groove for the radial nerve on the ectepicondyle and the ectepicondylar foramen is elongated, as the short supinator process is transversely narrow like in all “younginiform” reptiles and is nearly confluent with the humeral shaft, thereby failing to enclose the ectepicondylar groove like in *Youngina capensis* (Gow, 1975) and *Acerosodontosaurus piveteaui* (Bickelmann et al., 2009). The transverse width of the entepicondyle is roughly half its distal transverse width like that of all “younginiform” reptiles. The angle of the proximal margin of the entepicondyle relative to the main axis of the humerus is also obtuse, as the entepicondyle curves smoothy into the diaphysis and the distal extension terminates proximal to the distal margin of the trochlea. This condition is consistent with all known “younginiform” reptiles except *Hovasaurus boulei* (Currie, 1981).

The radius and ulna are exceedingly slender bones of the forelimb, measuring roughly 30% shorter than the humerus and are subequal to each other in length, unlike that of *Youngina capensis* where the radius is considered slightly longer (Gow, 1975) (Figs. 7, 8, S6). Rather interesting, the ulna appears to have only a modestly developed proximal expansion and a poorly developed olecranon like that of *Saurosternon bainii* (Carroll, 1975) and is lower than that of *Youngina capensis* (Gow, 1975). The radius is also twisted slightly along its length while the ulna is straight along its length.

The carpus is best represented by the μCT rendering of the right forelimb of SAM-PK-K7710a (Fig. S6) and is largely disarticulated in SAM-PK-K7710c and SAM-PK-K7710e (Fig. 8). The maximum width of the carpus excluding the pisiform is less than the length of metacarpal IV and the ulnare is short. The length of metacarpal IV is greater than half that of the radius and subequal to that of metacarpal III. A carpal intermedium, distal carpal one, and distal carpal five are also present. The manual formula is 2-3-4-5-3 like that of all other “younginiform” reptiles and each digit has short, distally tapering unguals with a slight ventral recurvature, each subequal or shorter than the preceding phalanx. The penultimate phalanx is also shorter than the preceding phalanx.

The pelvis is exposed in SAM-PK-K7710b and SAM-PK-K7710d-e (Figs. 7, 8, S5). SAM-PK-7710b clearly shows a thyroid fenestra of the puboischiatic plate and it is bound ventrally by a strong pubo-ischium suture. The thyroid fenestra is indeed present in *Youngina capensis* (Gow, 1975) and possibly *Saurosternon bainii* (Carroll, 1975). A thyroid fenestra is typical of many lepidosauromorphs as well as some archosauromorphs like tanystropheids (Spiekman et al., 2021). The acetabulum is irregularly shaped and bounded by a prominent anterodorsal bony lamina framing the anterodorsal margin. The ilium does not possess a pubic flange, and the iliac blade is elongated and slender, extending three times its maximum dorsoventral height in the posterodorsal direction past that the posterior margin of the ischiatic peduncle. It is also less vertically oriented than the broad, vertically oriented iliac blade of *Youngina capensis* (Gow, 1975) and more closely resembled that of *Acerosodontosaurus piveteaui* (Bickelmann et al., 2009), *Claudiosaurus germaini* (Carroll, 1981) and earlier diverging diapsids like *Araeoscelis gracilis* (Reisz, 1981). The ischium is also rather typical of *Youngina capensis* (Gow, 1975) and the posterior margin of the ischium is vertical and flattened. Interestingly, there is a pubic tubercle contrary to that of *Youngina capensis* (Gow, 1975), although its size and shape are difficult to determine*.*

The femur is represented in all individuals of the SAM-PK-K7710 except for SAM-PK-K7710c (Figs. 7, 8). It is a long, unusually slender, and slightly sigmoidal hindlimb bone articulating to the pelvis, tibia, and fibula. The femur is also roughly subequal in length to the tibia and fibula, a condition also observed in *Saurosternon bainii* (Carroll, 1975), *Youngina capensis* (Gow, 1975), and the lepidosauromorph *Palaeagama vielhauri* (Carroll, 1975). The proximal surface is well-ossified and rounded and this tuberosity may represent a distinct epiphysis but this is difficult to confidently determine. The well-developed internal trochanter is nearly at the same level as the proximal articulating surface and is separated from the proximal head by a slight concavity. There is no evidence of a fourth trochanter and little or no adductor ridge on the shaft. The distal condyles are also subequal and show limited expansion beyond the circumference of the femoral shaft. The medial surface of the tibial condyle is convex and rounded, as is the lateral surface of the femoral condyle, while the ventral surface of the fibular condyle is flat.

The tibia is represented in all individuals of SAM-PK-K7710 except SAM-PK-K7710c (Figs. 7, 8). It is a straight, long, and slender bone with a small proximal head that is reduced compared to other stem saurians and it lacks the typical proximal crest on the margin of the posteromedial shaft seen in lizards, just as in *Saurosternon bainii* (Carroll, 1975). The distal expansion to receive the calcaneum is also surprisingly modest.

The fibula is visible in SAM-PK-K7710a-b and SAM-PK-K7710e in articulation with the rest of the hindlimb (Figs. 7, 8). It is a very slender, straight, and elongated bone of the hindlimb articulating distally with the proximal surfaces of the astragalus and calcaneum. It is similar in length to the tibia but is significantly more slender.

**Fig. 12:**
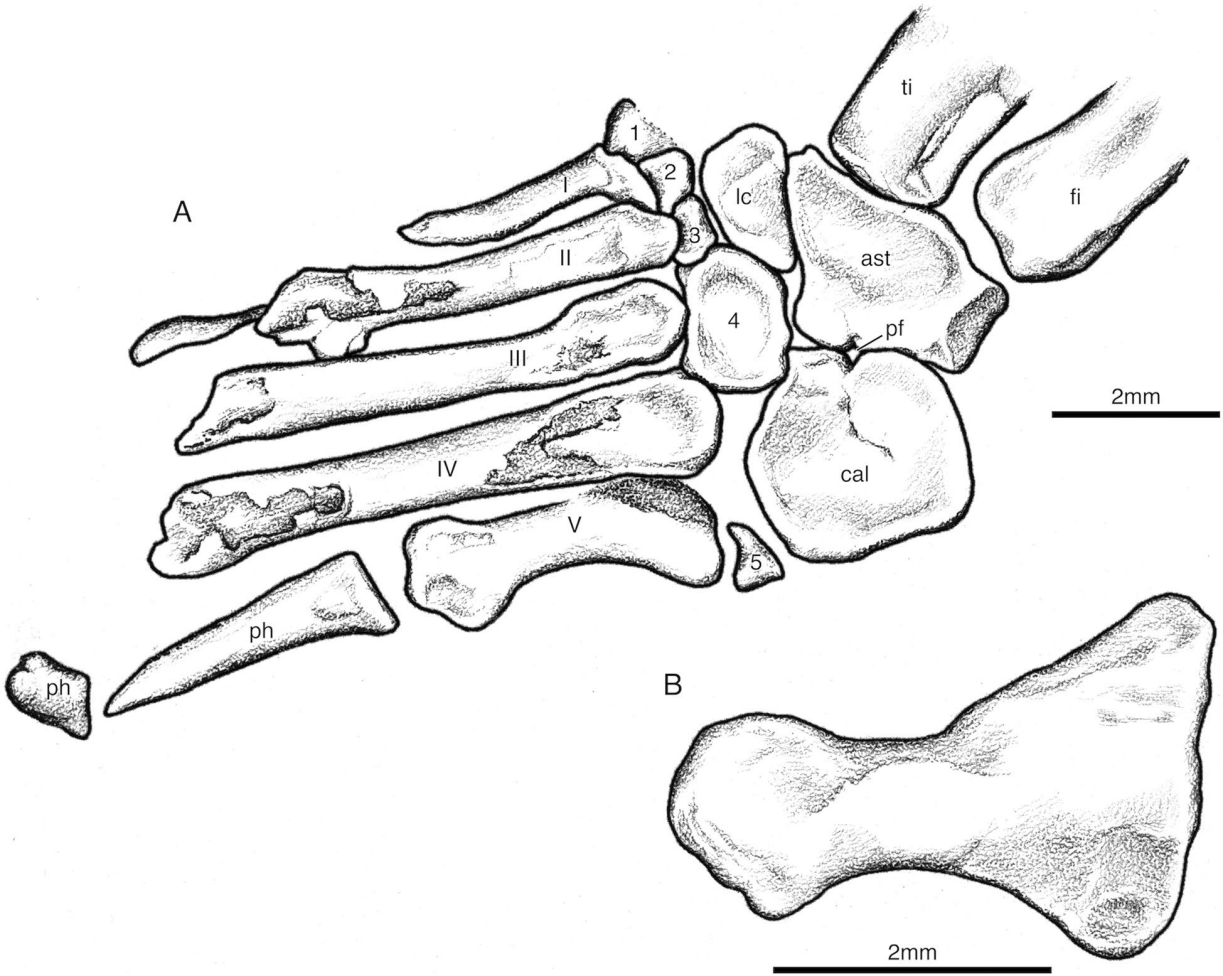
*Akkedops bremneri* SAM-PK-K7710 pes illustrations. **A** SAM-PK-K7710a left pes in dorsal view. **B** SAM-PK-K7710b left metatarsal V dorsal view. *ast* astragals, *cal* calcaneum, *lc* lateral centrale, *pf* perforating foramen, *ph* phalange, *1–5* tarsals, *I-V* metatarsals

The tarsus is best represented in SAM-PK-K7710a-b and SAM-PK-K7710d but also as partial and disarticulated bones in SAM-PK-K7710e (Figs. 7, 8, 12A). Important similarities can be made with *Saurosternon bainii*, and *Palaeagama vielhauri* (Carroll, 1975) since the pes of *Youngina capensis* is poorly known. The pes of *Akkedops bremenri* is significantly longer than the manus and the transverse width of the tarsus at the widest point is less than the length of metatarsal IV. The astragalus is subtriangular and the proximal neck region is long and it does not possess a posterior groove. A distinct ridge also separates the tibial and fibular facets, which grade gently into an anterior depression. Like *Saurosternon bainii*, the astragalus and calcaneum are not fused and a perforating foramen exists between them, although more so on the calcaneum and the calcaneum does not have a lateral tuber (Carroll, 1975). The metatarsals are positioned to diverge from the ankle and the metapodials overlap proximally like in *Saurosternon bainii* (Carroll, 1975). Metatarsal V is longer than half that of metatarsal III and it is proximally expanded like in many “younginiform” reptiles but differs from that of *Saurosternon bainii* in not being hook-shaped to hug the third distal tarsal (Carroll, 1975). Metatarsal V is rather large, lacks dorsiflexion, and is distinctly hook-shaped with a large proximal expansion as in *Saurosternon bainii* (Carroll, 1975) (Fig. 12B). Its proximal end possesses a prominent laterally pointed process which curves medially to contact the fourth tarsal, creating a hook-like outline like that of *Saurosternon bainii* and unlike *Palaeagama vielhauri* (Carroll, 1975). All five distal tarsals are present like in *Saurosternon bainii* and in *Youngina capensis* (Carroll, 1975; Gow, 1975). The transverse width of the fourth distal tarsal is wider than that of the third distal tarsal and its proximal contact is a smooth surface able to receive proximal tarsals. The lateral and medial distal articular portions of the distal non-ungual phalanges are subparallel and the proximodistal length of the penultimate phalanx is shorter than proceeding phalanx. The unguals are also subequal or shorter than the preceding phalanx and they lack a ventral tubercule.

### Phylogenetic analysis

**Fig. 13:**
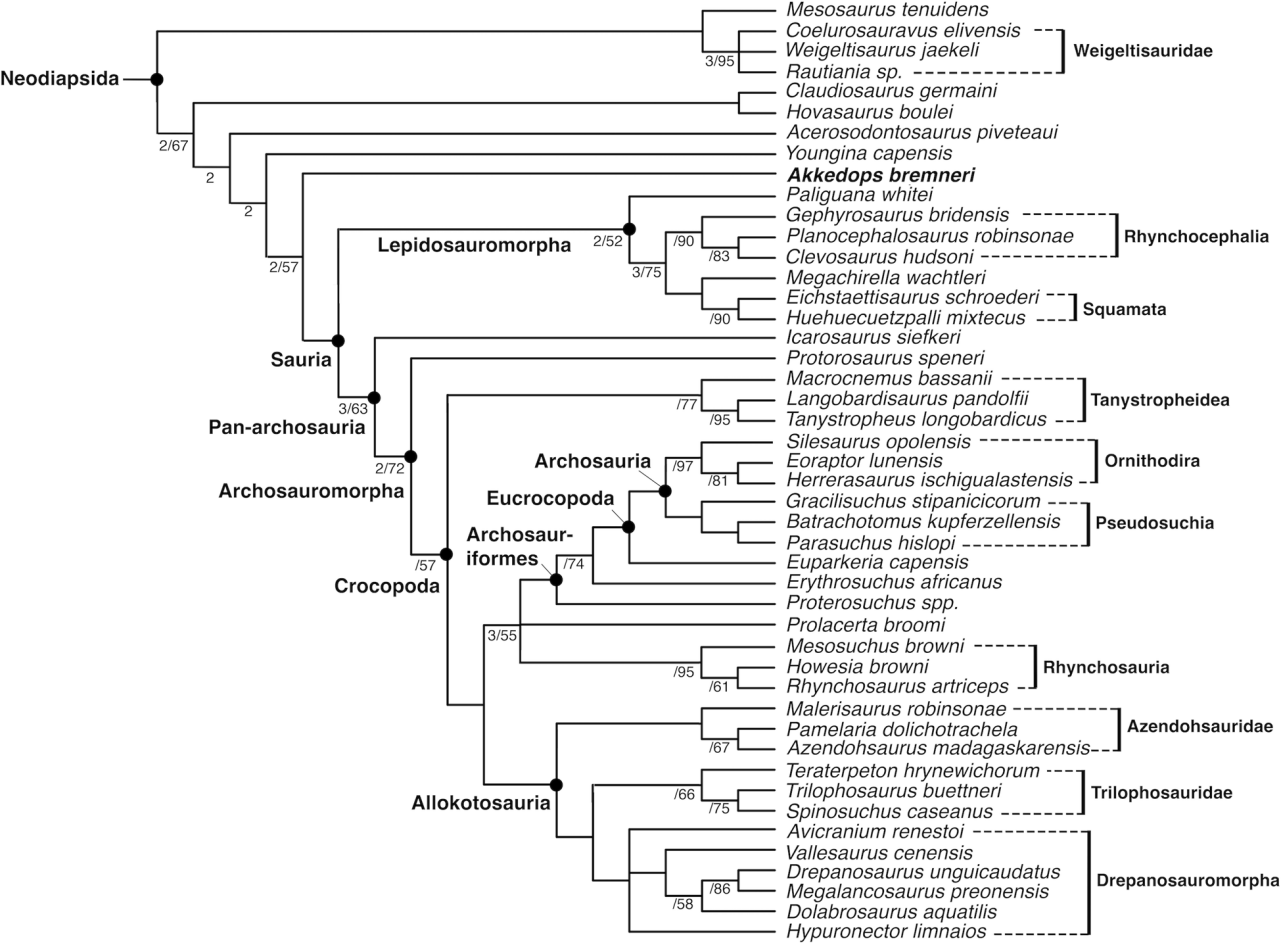
Early neodiapsid phylogeny. Strict consensus tree from Maximum Parsimony analysis (12MPTs of 2634 steps; CI 0.202; RI = 0.602). Node labels: Bremer value (when > 1)/Bootstrap (when > 50%). Full tree is available in Fig. S7

We included *Akkedops bremneri* in the most recently published data matrix on patterns of early diapsid evolution and diversification (Buffa et al., 2024). Our maximum parsimony analysis recovered 12 most parsimonious trees (MPTs) of 2634 steps with a consistency index (CI) of 0.202 and a retention index (RI) of 0.602 (Fig. 13).

We recover largely the same tree as Buffa et al. (2024), with a few impactful exceptions owing to changes in specific scorings of *Youngina capensis* after reexamination of the respective material and removal of SAM-PK-K7710 from that taxon (specific changes to the scoring of *Youngina capensis* are located in Supplementary File 1). The newly added *Akkedops bremneri* is recovered as sister to Sauria, while *Youngina capensis* is the sister taxon to the clade of *Akkedops bremneri* + Sauria. Our results do not support a monophyletic clade of the other “younginiform” reptiles *Acerosodontosaurus piveteaui* and *Hovasaurus boulei*, nor *Youngina capensis* for which SAM-PK-K7710 was previously referred to. *Claudiosaurus germaini* is also recovered outside of Weigeltisauridae and sister to *Hovasaurus boulei*.

## Discussion

### Relations of *Akkedops bremneri* and *Youngina capensis*

*Akkedops bremneri* and *Youngina capensis* are clearly distinct taxa and the evidence presented here indicates that they are positioned closely to one another along the saurian stem (Fig. 13). They share many common stem saurian anatomies, however, there are several clear differences and *Akkedops bremneri* can be differentiated from *Youngina capensis* by the following significant anatomical features.

Overall, the skull of *Akkedops bremneri* is roughly half the size of *Youngina capensis *and has a shorter, wider rostrum, larger orbits, while also lacking postparietals, tabulars, and a lower temporal bar (Figs. 1, 2, 3, 4). The nasals are half the anteroposterior length of the frontals and they are transversely broader to form a posterolaterally sloping medial suture with the frontal and prefrontal. Lateral exposure of the lacrimal is greatly reduced and it contributes little to the anterior orbital margin, which is compensated for by a more robust ventral process of the prefrontal. The maxilla is also dorsoventrally low and has twenty-six relatively small peg-like, non-serrated teeth with very slight recurvature at the crown apex (Figs. 2, 3, 4B, G).

There are also striking differences in the temporal architecture of *Akkedops bremneri*. The jugal has a greatly reduced free ending subtemporal process and the quadratojugal is substantially reduced and sutures to the quadrate, to form an open ventral margin of the lower temporal fenestra (Fig. S1). A strong lateral extension of the supratemporal contacts the posterior ramus of the postorbital, thereby contributing to the posterior margin of the upper temporal fenestra and effectively reinforcing the parietal-squamosal suture (Figs. 1A, B, 3A, B, 4A–G). The squamosal is also elongated and rather slender with only minimal dorsal expansion (Figs. 1A, B, 2A, B, 3A, B, 4A–D). The quadrate is especially distinctive, possessing unique medial and posterior flanges, a tympanic crest, and is posteriorly emarginated (Figs. 2A, B, S2).

The palate of *Akkedops bremneri* is rather more dentulous with wide, densely populated denticle fields, particularly that of the palatine, which has denticles roughly twice as large as those of the pterygoid and vomer (Fig. S3). There is also a wide shelf of denticles spread across the ventral surface of the transverse flange of the pterygoid. The vomers are also shorter to complement the anteroposteriorly short rostrum and the anterior process terminates at a single point.

The braincase is very similar to *Youngina capensis* except the distal ends of the exoccipitals are more expanded and the anterior extension of the cultriform process is also relatively short (Figs. 1C, D, S4). The mandible also appears rather similar except that the lateral exposure of the surangular is greater than that of the angular (Figs. 2A, B, 3C, D).

There are also several differentiating features of the postcranial skeleton, specifically those of the limbs and the vertebrae. All presacral vertebrae have two distinct costal facets, the pedicel is dorsoventrally taller than the respective centrum, and the transverse breadth of the neural arch excluding the transverse processes is broader than the respective centrum, and the iliac blade is comparatively more slender and less vertically oriented (Figs. 9C, 10, S5). The proportions of the forelimb also differ significantly from that of *Youngina* *capensis* as discussed in the description (Figs. 7, 8).

### Implications for stem saurian evolution and diversification

*Akkedops bremneri* is an unusual neodiapsid in several respects and shows a combination of features suggestive of both stem saurian and saurian affinities, and is recovered in our analysis as sister to Sauria. *Akkedops bremneri* displays the following unambiguous saurian synapomorphies outlined in Buffa et al. (2024): pineal foramen less than 25% of the interparietal length, postparietals absent, a quadrate that is posteriorly emarginated and has a moderate lateral flange of the quadrate (tympanic crest), the basioccipital tubera are well-developed and extend ventral to the occipital condyle, lack of a posterior groove of the astragalus, the proximal end of metatarsal V is hook-shaped where the proximal process gradually curves medially, the maximum width of the carpus excluding the pisiform is less than the length of the fourth metacarpal, and the height of the coronoid process is shorter than that of the anterior process of the jugal.

Additionally, several skeletal features typical of more early diverging neodiapsids are also present. This includes the presence of a retroarticular process that is dorsoventrally shallow with its dorsal margin posteroventral to the quadrate articulation in lateral view, the absence of an anterior surangular foramen, a fifth distal carpal and distal tarsal, and the proximodistal length of metatarsal V is slightly longer than half that of metatarsal III. There is also no cephalic condyle or dorsal expansion of the dorsal head of the quadrate and the palatal bones are extensively covered by small teeth, some of which are even present on the base of the cultriform process. This mosaic of more primitive and derived features suggests a surprising complexity surrounding the early evolution of stem saurians, even in the absence of abundant and comparably complete skeletal materials for this early stage of diapsid evolution.

Our anatomical reevaluation of *Youngina capensis* and inclusion of *Akkedops bremneri* in this phylogenetic analysis by Buffa et al. (2024) greatly clarifies the positions of several Permian neodiapsids along the saurian stem. We recover *Youngina capensis* closer to Sauria outside of a clade with *Acerosodontosaurus piveteaui* and *Hovasaurus boulei* and that *Claudiosaurus germaini* is sister to *Hovasaurus boulei* outside of Weigeltisauridae. As such, we provide additional evidence that taxa often considered “younginiform” reptiles (e.g., Buffa et al., 2024; Currie, 1982; Evans, 1988; Laurin, 1991; Simões et al., 2022) do not form a monophyletic clade and it appears that they grade into Sauria, as has also been proposed by other studies (e.g., Bickelmann et al., 2009; Reisz et al., 2011).

This phylogenetic context in which we describe *Akkedops bremneri* as a new stem saurian, also provides critical insights into the apparent emergence of the general lepidosauromorph body plan along the saurian stem and other anatomies of potential relevance to the origin of Sauria. There are several lepidosaur-like features of the postcranial skeleton demonstrated by late Permian neodiapsids, particularly *Saurosternon bainii*, and now *Akkedops bremneri* (Carroll, 1975). It is important to emphasize that while relatively more complete skeletons of the highly autapomorphic semiaquatic neodiapsid taxa (e.g., *Acerosodontosaurus piveteaui* [Bickelmann et al., 2009], *Hovasaurus boulei* [Currie, 1981], *Claudiosaurus germaini* [Carroll, 1981]) are somewhat common, articulated skeletons of terrestrial neodiapsids are exceedingly rare. All currently recognized terrestrial have postcranial skeletons with general proportions appearing to favor small, gracile, lizard-like body plans with delicate long hind limbs, relatively shorter but still slender forelimbs, and especially long and slender tails, overall resembling that of the lepidosauromorph *Palaeagama vielhauri* (Carroll, 1975). The straight transverse processes of the first five caudal vertebrae in the absence of caudal ribs, exemplified by *Akkedops bremneri* and many other "younginiform" reptiles, but also millerettids (Gow, 1972), is al﻿so observed in several early diverging saurians (Carroll, 1975; Ezcurra et al., 2014) and may represent a typical condition of many Paleozoic neodiapsids and some early diverging saurians. The mild lateral embayment of the dorsal margin of the upper temporal fenestra and the depressed shelf of the posterolateral margin of the ventral ramus of the postorbital are also observed in various Paleozoic neodiapsids and early diverging saurians. Also worth noting is the presence of a thyroid fenestra in *Akkedops bremneri* and *Youngina capensis*, a feature more typical of many lepidosauromorphs and some archosauromorphs, as well as the potential secondary epiphyseal ossifications of the humerus and femur.

Clearly, the early evolution of many features considered more typical of early diverging saurians is complex and poorly understood, many of which are likely to be clarified by new Permian neodiapsids. Given that the early stages of diapsid evolution took place in the shadow of the ever dominant synapsids and parareptiles of the late Paleozoic terrestrial ecosystems, it is likely that this pattern influenced their evolution and diversification into predominantly small gracile predatory and insectivorous niches, many of which were also likely nocturnal. These new skeletons of *Akkedops bremneri* are an exceptional resource for understanding the early stages of neodiapsid evolution along the saurian stem and are thus of great significance to future investigations into the origin of this large and prolific clade.

## Supplementary Information

Additional file 1.

Additional file 2.

## Data Availability

All data generated or analyzed as part of this study are available. μCT scan data for SAM-PK-K6205 and SAM-PK-K7710 are available on MorphoSource (http://morphobank.org/permalink/?P5553) and all additional phylogenetic data can be found in the supplementary information files.
