# Supplementary material for: A new stem saurian reptile from the late Permian of South Africa and insights into saurian evolution
Source: Swiss J Palaeontol. 2025 Feb 26;144(1):10. doi: 10.1186/s13358-025-00351-y (PMC11865139; doi:10.1186/s13358-025-00351-y)
Supplement: Supplementary file 2 — Additional file 2. [file 13358_2025_351_MOESM2_ESM.pdf]

Supplementary File

A new stem saurian reptile from the late Permian of South Africa and insights into early saurian evolution

Ethan Dean Mooney[1,2*], Diane Scott[1], Robert Raphael Reisz[2,1*]

[1]Department of Biology, University of Toronto Mississauga, 3359 Mississauga Rd., Mississauga L5L1C6, Ontario, Canada

[2]Dinosaur Evolution Research Center, International Center of Future Science, Jilin University, 2699 Qianjin Str., Changchun, Jilin Province 130012, China

Several character states for *Youngina capensis* were reassessed and changed upon reexamination of *Youngina capensis* material and removal of SAM-PK-K7710 from that taxon. A total of 10 character states were changed for *Youngina capensis* from Buffa et al. (2024), which include: character #67 0→1 the postfrontal size is approximately less than half the size of postorbital; character #77 1→0 supratemporal bar positioned distinctly ventral to dorsal border of orbit broadly exposing supratemporal fenestra in lateral view; character #78 change ?→1 infratemporal bar narrow less than 20% skull height; character #80 change 1→2 jugal anterior process higher than suborbital process of maxilla and with an ascending subprocess; character #159 change 0→1 opisthotic paroccipital process anteroposteriorly flattened distally; character #186 change ?→1 mandible retroarticular process formed only by articular; character #296 change ?→0 scapulacoracoid both bones fuse with each other in mature individuals present; character #297 change ?→1 scapulocoracoid notch on anterior margin at level of suture between both bones present; character #311 change 1→? sternal plates are unknown; character #343 change 0→1 thyroid fenestra is present and small.

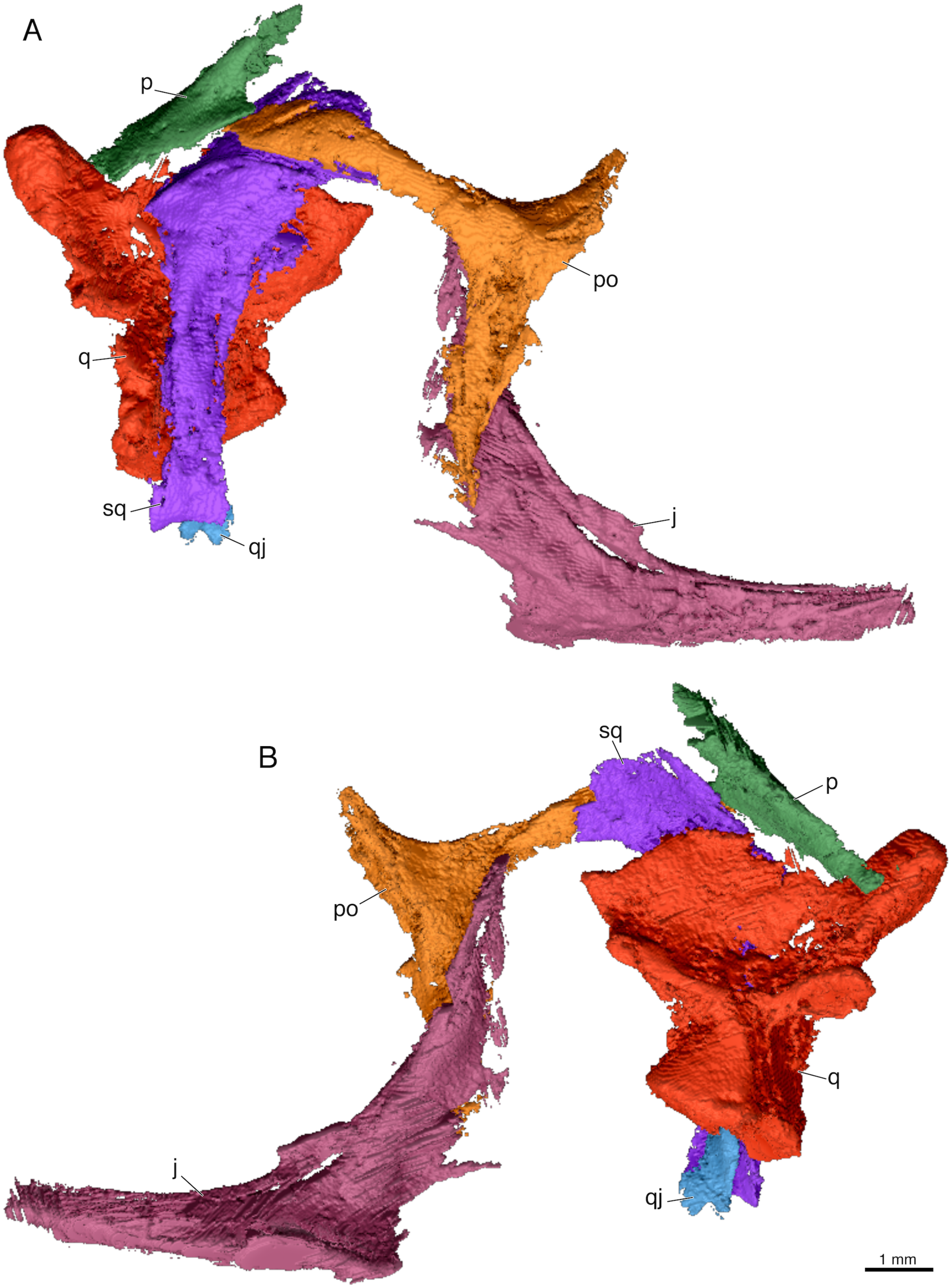

Fig. S1 *Akkedops bremneri* holotype skull SAM-PK-K6205 µCT rendering of temporal region. **A** right lateral view. **B** right medial view. *j* jugal, *p* parietal, *po* postorbital, *q* quadrate, *qj* quadrotojugal, *sq* squamosal.

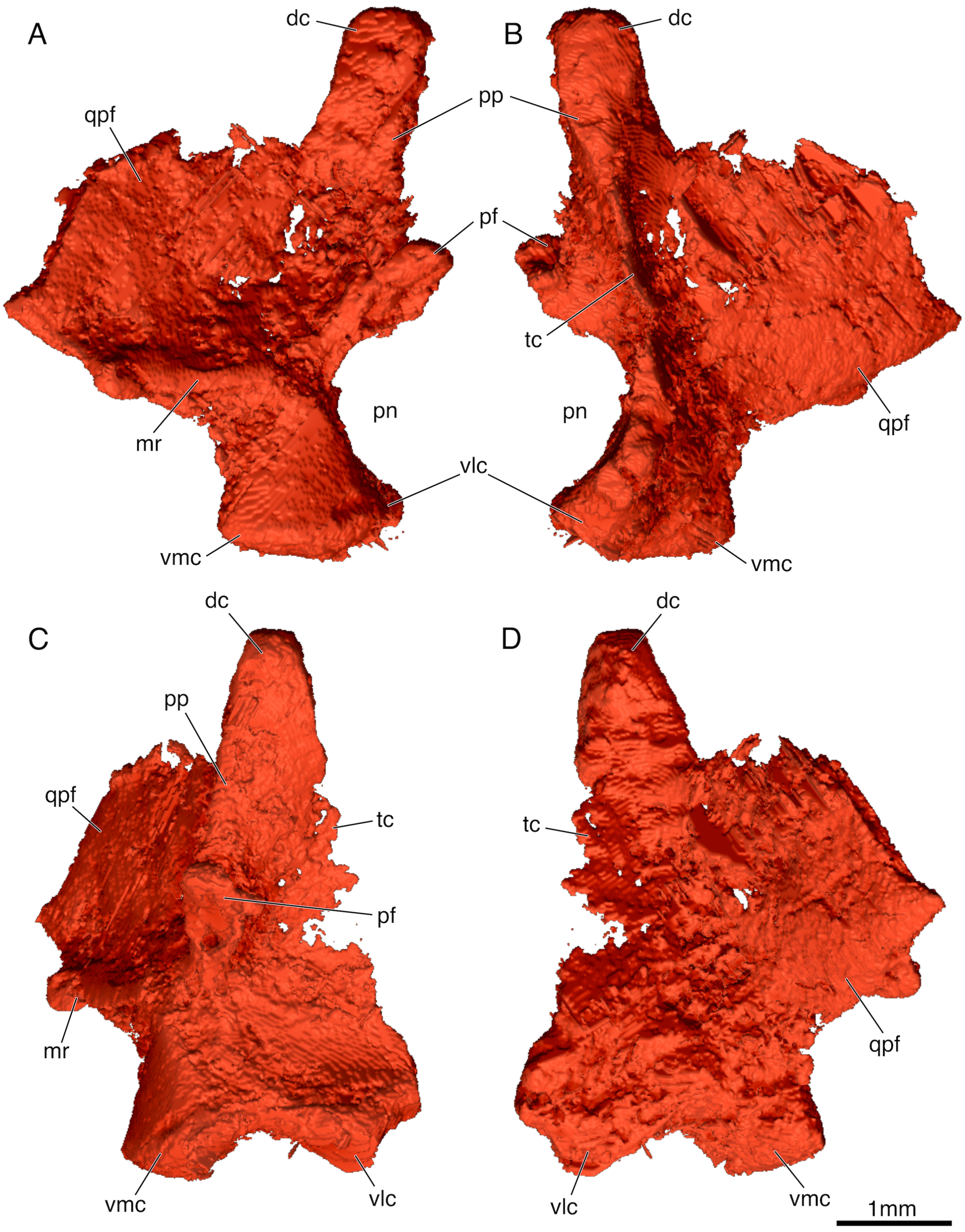

Fig. S2 *Akkedops* SAM-PK-K6205 right quadrate. **A** medial view. **B** lateral view. **C** posterior view. **D** anterior view. *dc* dorsal condyle, *mr* medial ridge, *pf* posterior flange, *pn* posterior notch, *pp* posterior pillar, *qpf* quadrato-pterygoid flange, *tc* tympanic crest, *vlc* lateral condyle, *vmc* medial condyle.

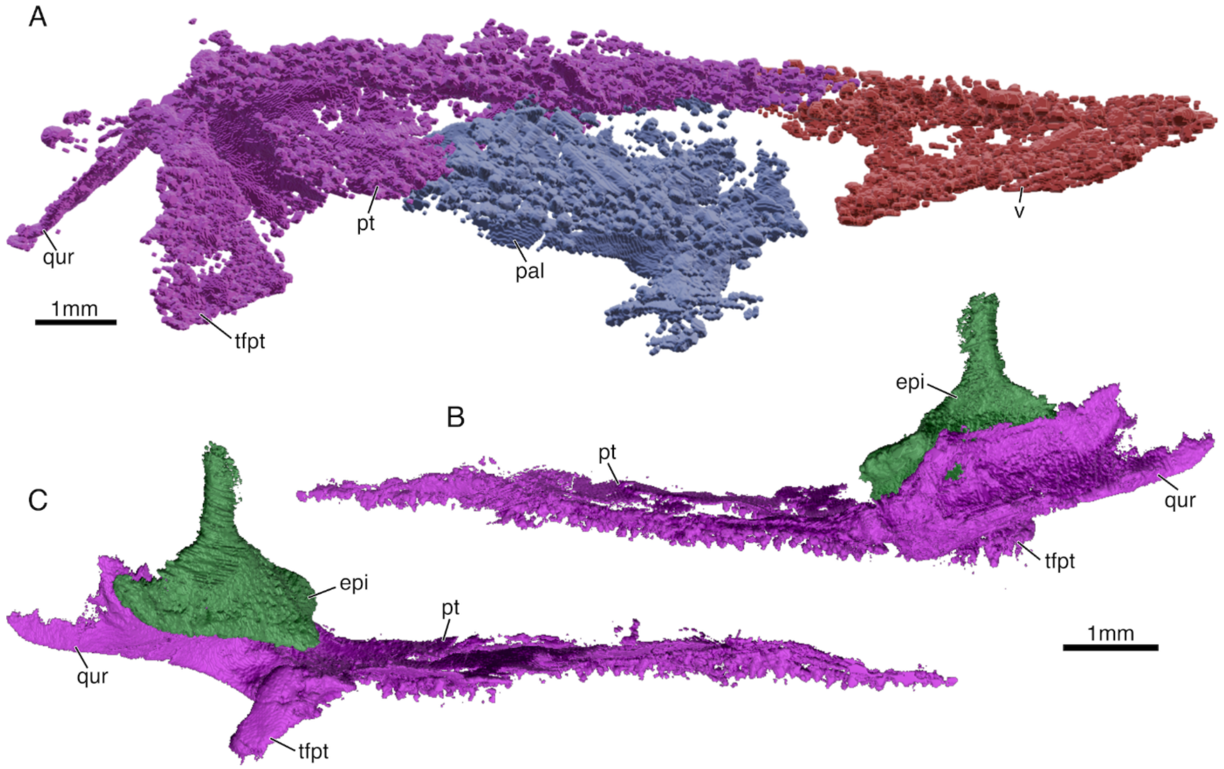

Fig. S3 *Akkedops bremneri* SAM-PK-K6205 µCT rendering of palatal elements. **A** rearticulated palate excluding ecopterygoid in ventral view. **B** pterygoid and epipterygoid in medial view. **C** pterygoid and epipterygoid in lateral view. *epi* epipterygoid, *pal* palatine, *pt* pterygoid, *qur* quadrate ramus of pterygoid, *tfpt* transverse flange of pterygoid, *v* vomer.

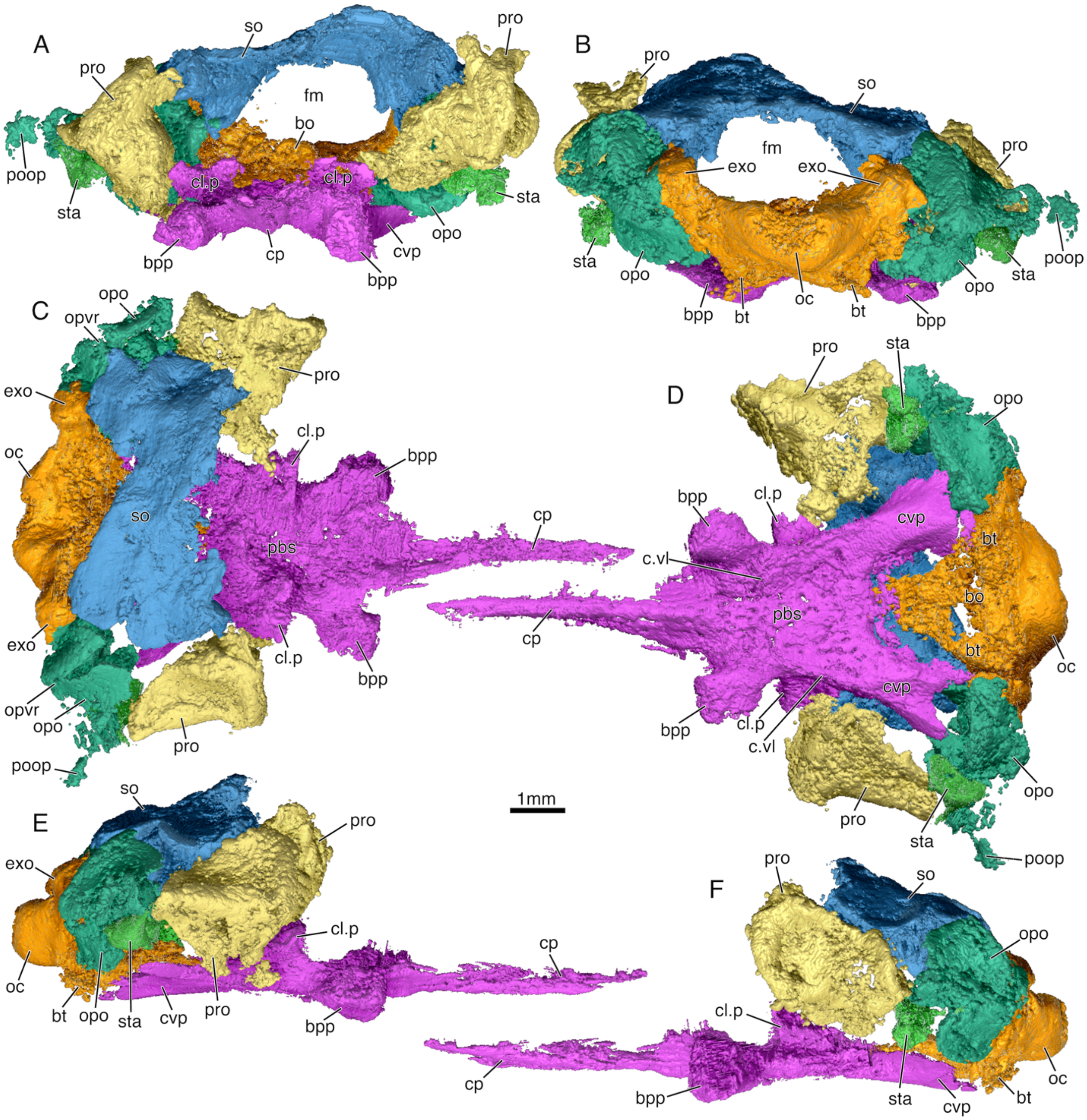

Fig. S4 *Akkedops bremneri* holotype SAM-PK-K6205 neurocranium µCT rendering. **A**, anterior view. **B** posterior view. **C**, dorsal view. **D** ventral view. **E**, right lateral view **F** left lateral view. *bo* basioccipital, *bpp* basipterygoid process, *bt* basal tubera of basioccipital, *c.vl* crista ventrolateralis, *cl.p* clinoid process, *cp* cultriform process, *cvp* crista ventrolateralis process of the parasphenoid, *exo* exoccipital, *fm* foramen magnum, *oc* occipital condyle, *opo* opisthotic, *opvr* opisthotic ventral ramus, *pbs* parabasisphenoid, *poop* paroccipital process of the opisthotic, *pro* prootic, *so* supraoccipital, *sta* stapes.

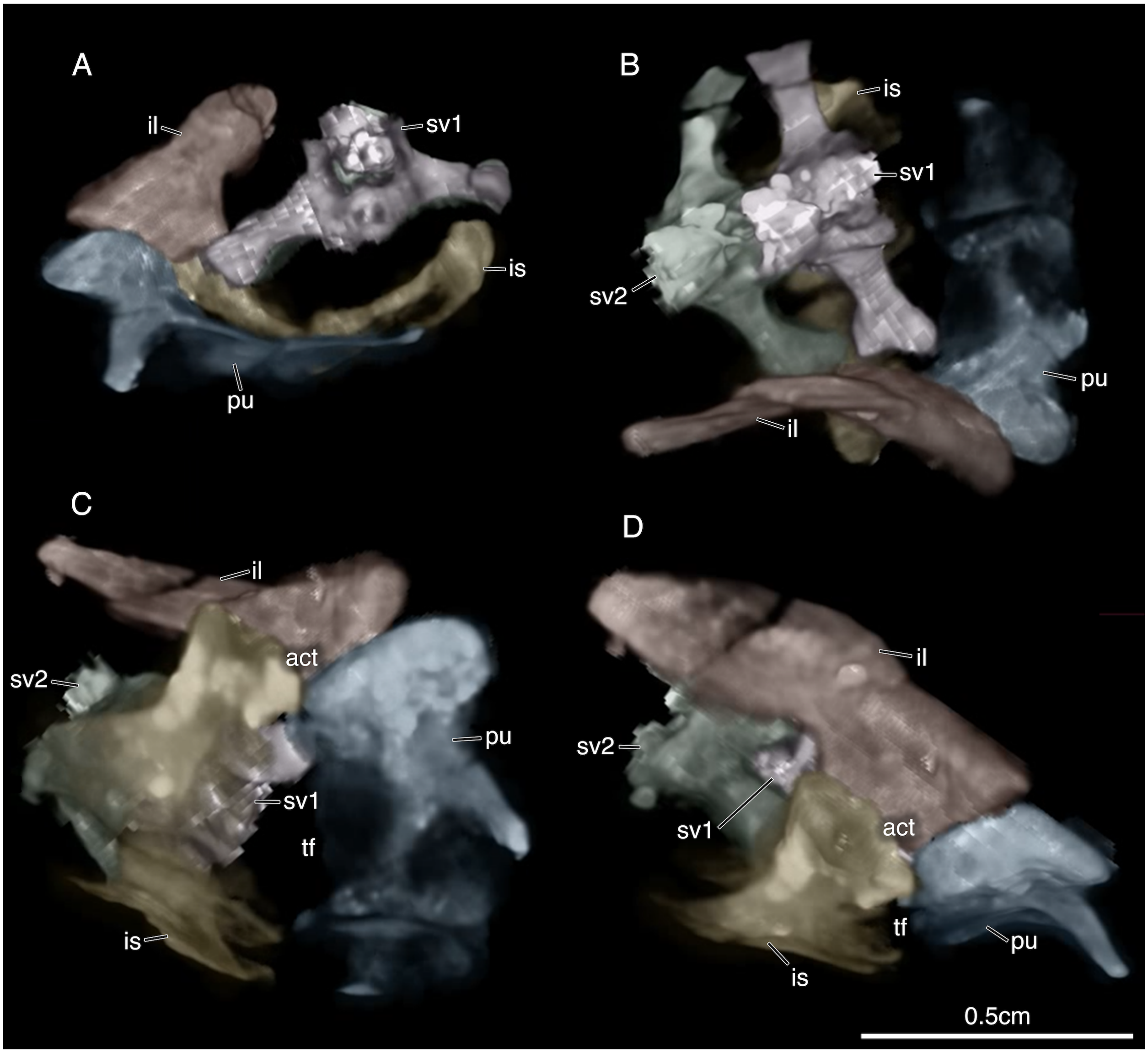

Fig S5. *Akkedops bremneri* SAM-PK-K7710e pelvis µCT rendering. **A** posterior view. **B** dorsal view. **C** left ventrolateral view. **D** left lateral view. *act* acetabulum, *il* ilium, *is* ischium, *pu* pubis, *sv* sacral vertebra, *tf* thyroid fenestra.

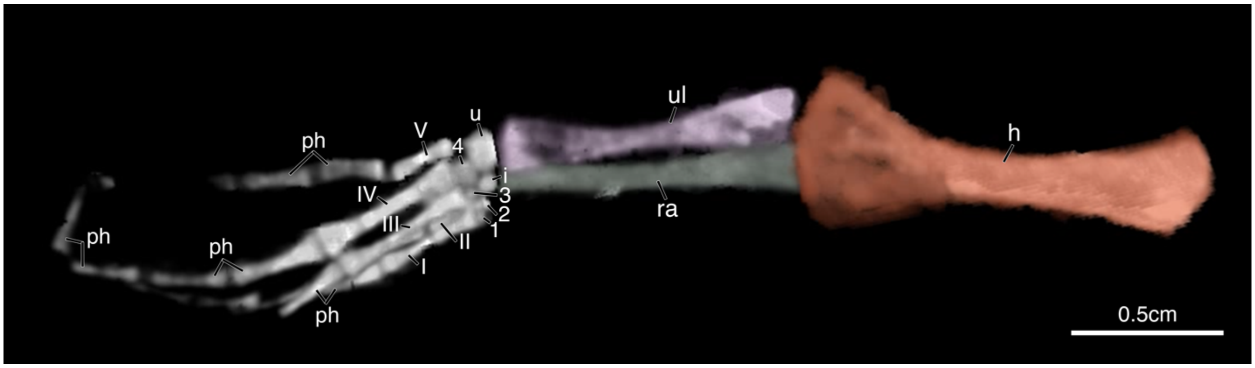

Fig. S6 *Akkedops bremneri* SAM-PK-K7710a right forelimb µCT rendering. *h* humerus, *i* intermedium, *ph* phalange, *ra* radius, *u* ulnare, *ul* ulna, *1-5* carpals, *I-V* metatacarpals.

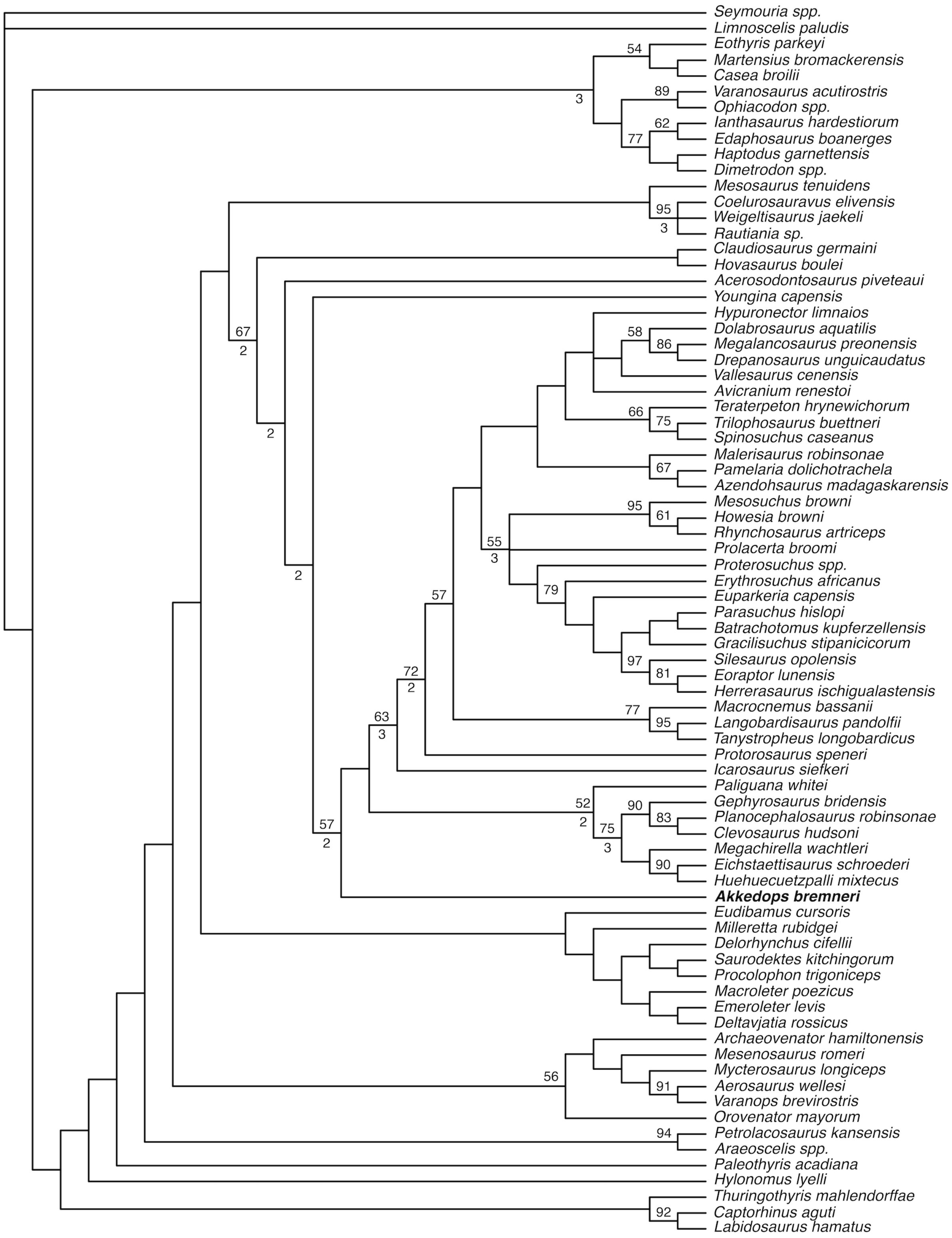

Fig. S7 Complete phylogeny from Fig. 13. Strict consensus tree from Maximum Parsimony analysis (12MPTs of 2634 steps; CI = 0.202; RI = 0.602). Node labels: Bremer value (when >1, below the branches) and Bootstrap frequencies (when >50%, above the branches).
